# X-ray-to-visible light-field detection through pixelated colour conversion

**DOI:** 10.1038/s41586-023-05978-w

**Published:** 2023-05-10

**Authors:** Luying Yi, Bo Hou, He Zhao, Xiaogang Liu

**Affiliations:** 1 Department of Chemistry, National University of Singapore, Singapore, Singapore; 2 Joint School of National University of Singapore and Tianjin University, Fuzhou, China; 3 Center for Functional Materials, National University of Singapore Suzhou Research Institute, Suzhou, China; 4 Institute of Materials Research and Engineering, Agency for Science, Technology and Research, Singapore, Singapore

**Keywords:** Imaging and sensing, Nanophotonics and plasmonics

## Abstract

Light-field detection measures both the intensity of light rays and their precise direction in free space. However, current light-field detection techniques either require complex microlens arrays or are limited to the ultraviolet–visible light wavelength ranges^1–4^. Here we present a robust, scalable method based on lithographically patterned perovskite nanocrystal arrays that can be used to determine radiation vectors from X-rays to visible light (0.002–550 nm). With these multicolour nanocrystal arrays, light rays from specific directions can be converted into pixelated colour outputs with an angular resolution of 0.0018°. We find that three-dimensional light-field detection and spatial positioning of light sources are possible by modifying nanocrystal arrays with specific orientations. We also demonstrate three-dimensional object imaging and visible light and X-ray phase-contrast imaging by combining pixelated nanocrystal arrays with a colour charge-coupled device. The ability to detect light direction beyond optical wavelengths through colour-contrast encoding could enable new applications, for example, in three-dimensional phase-contrast imaging, robotics, virtual reality, tomographic biological imaging and satellite autonomous navigation.

## Abstract (web summary)

Lithographically patterned perovskite nanocrystal arrays were used to determine radiation vectors from X-rays to visible light and the emission colours of the nanoparticles was used to create images of three-dimensional objects and for phase-contrast imaging.

## Main

Advances in materials and semiconductor processes have revolutionized the design and fabrication of micro- and nano-photodetectors. But the pixels of most sensors detect only the intensity of electromagnetic waves. As a result, all phase information of the objects and diffracted light waves is lost^5–10^. Although intensity information alone is sufficient for conventional applications such as two-dimensional photography and microscopy imaging, this limitation hinders three-dimensional (3D) and four-dimensional imaging applications, including phase-contrast imaging, light detection and ranging, autonomous vehicles, virtual reality and space exploration^11–19^. An optical array of microlenses or photonic crystals with pixelated photodiodes is usually used to measure the light field or the distribution of light directions and thus to characterize phase information. Nevertheless, integration of these elements into complementary metal-oxide-semiconductor architectures is costly and complex^4,20–22^. Optical resonances in subwavelength semiconductor structures enable the development of angle-sensitive structures by manipulating light–matter interactions^23–28^. However, most of them depend on wavelength or polarization and require materials with a high refractive index^29^. Moreover, light-vector detection and control are at present limited to ultraviolet- and visible-light wavelengths. Although several sensors using Shack–Hartmann or Hartmann structures are capable of phase measurements in the extreme ultraviolet-light range, phase measurements of hard X-rays and gamma rays remain challenging because high-energy beams cannot be focused using conventional mirrors or microlenses^30,31^.

**Fig. 1:**
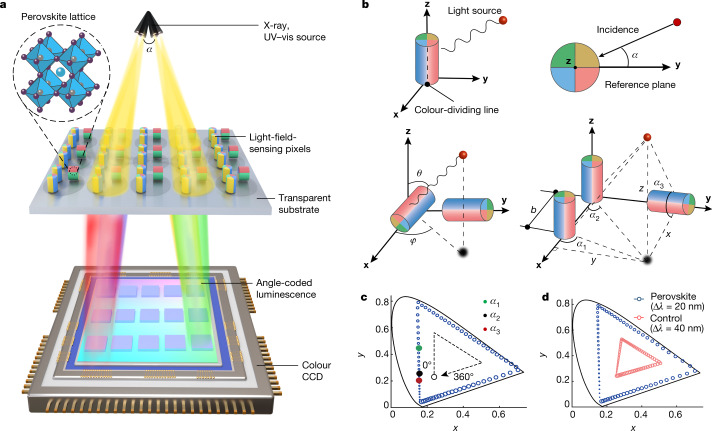
X-ray-to-visible light-field detection using pixelated perovskite nanocrystal arrays. **a**, Design of the 3D light-field sensor on the basis of pixelated colour conversion. Light-field-sensing pixels, which consist of patterned perovskite nanocrystals on a transparent film, convert light from different directions into luminescence signals of different colours, which can be detected by a colour CCD. UV–vis, ultraviolet–visible light. **b**, The working principle of light-field sensing by colour conversion. The basic unit of the 3D light-field sensor is a single azimuth detector comprising multicolour-emitting perovskite nanocrystals. The colour of output luminescence depends on the angle *α* between the incident light and the reference plane. Two perpendicularly arranged azimuth detectors can realize 3D light-field sensing and determine the azimuth angle *φ* and elevation angle *θ* of the incident light in spherical coordinates. By arranging three azimuth detectors, correlation of the three azimuth angles *α*_1_, *α*_2_ and *α*_3_ encoded in the colour outputs of the three azimuth detectors enables detection of the absolute position (*x*, *y*, *z*) of a light source. **c**, Chromaticity responses of a single azimuth detector at light incidence from 0° to 360° relative to the reference plane. Red, green and black dots correspond to the three azimuth angles *α*_1_, *α*_2_ and *α*_3_, respectively, recorded using the three azimuth detectors shown in **b**. **d**, Chromaticity response of a single perovskite nanocrystal-based azimuth detector at light incidence from 0° to 360°, relative to the control comprising ZnS:Cu^2+^/Mn^2+^ and SrAl_2_O_4_:Eu^2+^/Dy^3+^ phosphors.

Owing to the versatility of colour encoding in data visualization, we proposed that colour-contrast encoding could be used to visualize the directions of light rays. To test our hypothesis, we selected inorganic perovskite nanocrystals because of their excellent optoelectronic properties^32–35^. They also demonstrate highly efficient and tunable emission with high colour saturation across the visible spectrum under X-ray or visible-light irradiation. Furthermore, Sn-based perovskite nanocrystals can have optical bandgaps that extend into the near-infrared light region^36,37^. A fundamental design for a 3D light-field detection involves lithographically patterning perovskite nanocrystals onto a transparent substrate (Fig. 1a). A 3D light-field sensor can then be constructed by integrating the patterned thin-film substrate with a colour charge-coupled device (CCD) that converts the angle of incident light rays into a specific colour output. The basic unit of 3D light-field sensor is a single azimuth detector comprising multicolour-emitting perovskite nanocrystals. When incident light strikes patterned nanocrystals, the azimuth angle *α* between the incident light and the reference plane can be detected by measuring the colour output of the basic unit (Fig. 1b). Specifically, two azimuth detectors arranged perpendicular to each other can realize 3D light-direction sensing and determine the azimuth angle *φ* and elevation angle *θ* of the incident light in spherical coordinates. To determine the absolute position of a light source, three azimuth detectors can be arranged to create a correlation among the three corresponding azimuth angles *α*_1_, *α*_2_ and *α*_3_ encoded in the colour outputs.

As a proof of concept, we synthesized inorganic perovskite nanocrystals (CsPb*X*_3_; *X* = Cl, Br or I) according to the literature^36,38,39^ (Supplementary Information section 1). We selected three sets of perovskite quantum dots with emissions at 445 nm, 523 nm and 652 nm to construct a single azimuth detector. When light is incident from 0° to 360° relative to the reference direction, the detected colour gamut forms a large triangle on the Commission Internationale de l’éclairage (CIE) *xy* chromaticity diagram (Fig. 1c). The position of the colour output on the chromaticity diagram determines the incident angle of the light and a larger triangle indicates a higher angular resolution. We found that the colour gamut of azimuth detectors made of perovskite nanocrystals forms a larger triangle in the chromaticity diagram compared with detectors made of ZnS:Cu^2+^/Mn^2+^ and SrAl_2_O_4_:Eu^2+^/Dy^3+^ phosphors (Fig. 1d). Azimuth detectors generate a higher angular resolution, because of the broader colour coverage and higher colour saturation of perovskite nanocrystals.

**Fig. 2:**
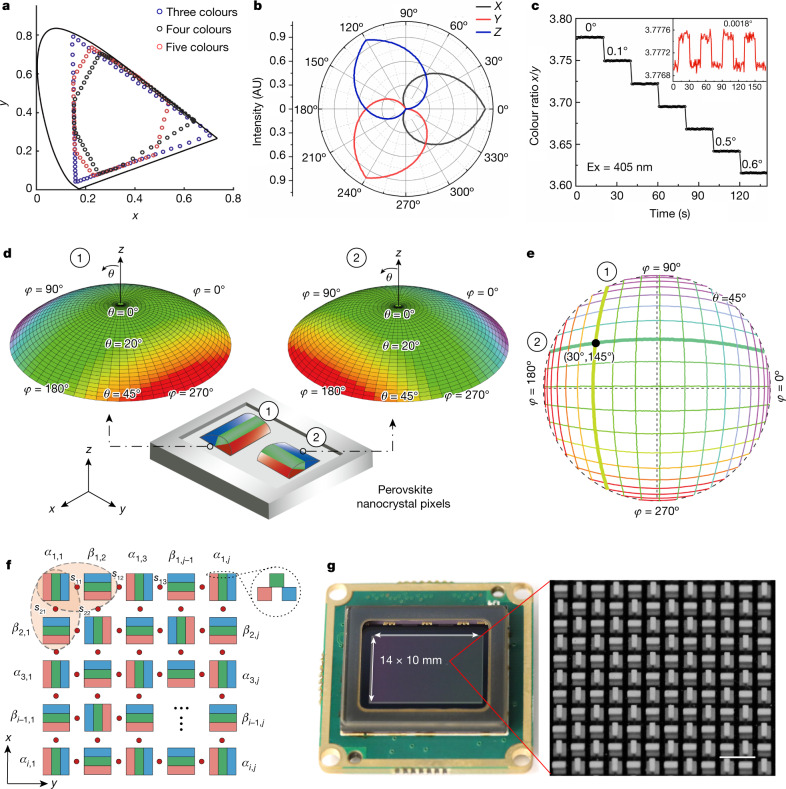
Characterization of the pixelated colour conversion for 3D light-field sensing. **a**, Chromaticity responses of single azimuthal detectors composed of three-, four- and five-colour perovskite nanocrystals as a function of the direction of incident light. **b**, CIE tristimulus values *X*, *Y* and *Z* of the output luminescence of a single azimuth detector as a function of the direction of incident light. AU, arbitrary units. **c**, Azimuth resolution measurement for visible light (405 nm) using a single azimuth detector, with a minimum detectable angular change of 0.0018°. Excitation (Ex) wavelength, 450 nm. **d**, Two types of colour map recorded from two perpendicularly aligned azimuth detectors with light incident from different azimuth angles *φ* and elevation angles *θ*. **e**, Contour lines extracted from the two colour maps in **d**. A unique incidence direction can be determined by combining the colour values from two azimuth detectors. **f**, Top view of the azimuthal detector arrays for imaging the 3D light direction, in which adjacent pixels of perovskite nanocrystals are aligned perpendicularly. The two detectors encircled by the ellipse can determine the angle of the beam incident on the centre point of the ellipse. Inset, side view of a patterned pixel. **g**, Photograph of a 3D light-field sensor fabricated by integrating the perovskite nanocrystal array into a colour CCD. Inset, a section of the microscopy image of nanocrystal-based azimuth detectors. Scale bar, 150 μm (**g**).

Single azimuth detectors with different colour gamuts produce colour plots of varying shapes (Fig. 2a). Nanocrystals with red, green and blue outputs can detect extremely small angular changes. We used this property and built a single three-colour azimuth detector on a red, green and blue sensor chip that converts incident light from 0° to 360° into different CIE *XYZ* tristimulus values of luminescence (Fig. 2b). The minimum detectable angular change is determined by the contrast ratio of the colour response and by the signal-to-noise ratio (SNR) of the colour sensor. Each primary colour in our tests contains 65,536 levels, which yields a detection limit of around 0.0018° angular change at a wavelength of 405 nm and a power of 8 mW (Fig. 2c).

We next designed and fabricated two azimuth detectors arranged perpendicular to each other for omnidirectional light-field detection (Fig. 2d). In spherical coordinates, the azimuth angle *φ* and elevation angle *θ* for each incident beam can be calculated using the formula *φ* = arctan(tan *α*_1_/tan *α*_2_) and *θ* = arctan[(tan^2^ *α*_1_ + tan^2^ *α*_2_)^1/2^], respectively, where *α*_1_ and *α*_2_ are obtained from the emission colours of the two azimuth detectors (Supplementary Information sections 2–4). The two azimuth detectors yielded two types of colour map at different angles of incidence. The contours of the two colour maps in the polar plot enable specific incidence angles to be determined by combining the colour values of two azimuth detectors (Fig. 2e). We further designed azimuthal detector arrays to image the 3D light direction, in which adjacent pixels of perovskite nanocrystals were aligned perpendicular to each other (Fig. 2f). For simplicity, the angle detected by detectors parallel to the *x* axis is denoted by *α*_*i*,*j*_ (where *i* and *j* refer to the rows and columns of the nanocrystal arrays); the angle detected by detectors parallel to the *y* axis is denoted by *β*_*i*,*j*_. Each of the two azimuth detectors, which are perpendicular to each other, can reconstruct the angle of the beam incident at the centre of the two pixels. For example, *α*_1,1_ and *β*_1,2_ can be used to calculate the 3D angle of the beam incident at point *s*_11_, whereas *β*_2,1_ and *α*_1,1_ can be used to calculate the 3D angle of the beam incident at point *s*_21_. Therefore, the imaging spatial resolution of the nanocrystal arrays is determined by the distance between *s*_11_ and *s*_12_. We next integrated a thin film of perovskite nanocrystal arrays into a digital camera equipped with a colour CCD (Fig. 2g). The CCD has a photosensitive area of 10 mm × 14 mm and a pixel size of 2.5 μm^2^ × 2.5 μm^2^; the pixel size of a single azimuth detector is 50 μm^2^ × 50 μm^2^.

**Fig. 3:**
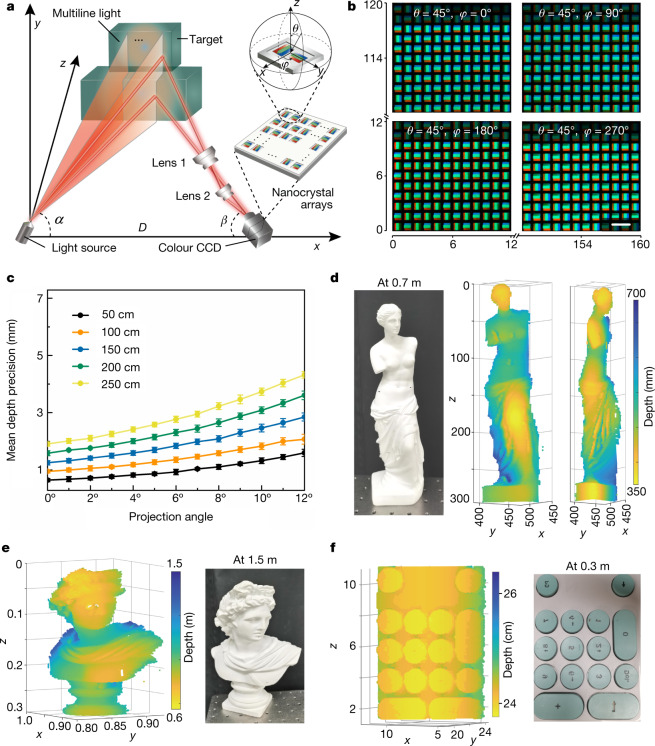
Three-dimensional imaging of real scenes by pixelated colour conversion. **a**, Schematic of the experimental set-up. Multiline structured light is incident on the object; lens 1 and lens 2 capture the reflected light and pass it to perovskite nanocrystal arrays. A colour CCD then measures the colour of each azimuth detector to calculate the corresponding distance to the scenes. **b**, Representative images of perovskite nanocrystal arrays with incident light from different directions. **c**, Mean depth precision plotted as a function of scene depth and radial position in the field of view. A movable, flat, white screen is used as the target object. Ten measurement trials were made for each projection angle and 20 measurement trials were performed for each depth. Data are mean ± s.e.m. **d**,**e**, 3D images of scenes placed at 0.7 m and 1.5 m. **f**, 3D depth image of a keyboard captured using the 3D light-field sensor. The colour map indicates the distance from the imaging point to the *z* axis at the origin (*x* = 0, *y* = 0). Scale bar, 150  μm (**b**).

A direct application of the light-field sensor based on pixelated perovskite nanocrystal arrays is 3D imaging and light detection and ranging (Fig. 3a). This imaging system is based on a triangulation method and consists of a multiline structured light source, two lenses for light collection and a colour CCD coated with a thin film of nanocrystal arrays. The object distance *z* is determined by measuring the angle of the light reflected to the nanocrystal arrays by the object—that is, a high angular resolution provides a high depth resolution. For a given pixel size (50 μm^2^ × 50 μm^2^), the theoretical depth resolution and detectable range are improved by about 10 times and 3 times, respectively, compared with conventional triangulation methods (Supplementary Information sections 5–7). To improve data accuracy, these nanocrystal arrays were calibrated first and then the imaging system was calibrated (Fig. 3b and Supplementary Information sections 8 and 9). Under light incidences from different angles *θ* and 𝜙, images captured using a perovskite nanocrystal array serve as a corresponding map of the colour response of each azimuth detector and the angle of incident light. To quantitatively evaluate the imaging performance of our prototype, we measured its depth accuracy as a function of scene depth and radial position within the field of view (Fig. 3c). These measurements showed an optimal depth accuracy of approximately 0.5 mm at a distance of 0.5 m, although the depth accuracy slightly decreased to approximately 1.5 mm at a distance of 2 m. Detector depth accuracy is affected by the power and angle of incident light. To ensure a high angular resolution, the power of the structured light source must be sufficient. The depth accuracy also varies depending on the intensity of the background light when the detector is used in natural-light or artificial-light conditions (Supplementary Information section 10). The dimensions of objects imaged by the light-field sensor at different distances (0.7 m and 1.5 m) are in accordance with their actual dimensions (Fig. 3d,e). Image reconstruction is also possible for objects with fine structures, such as keyboards and combs (Fig. 3f and Supplementary Information section 11). Insufficient light returned or random noise may result in undetected pixels. Moreover, we obtained 3D images of several objects of varying colours, sizes and materials at increasing depths through pixelated colour conversion (Supplementary Information section 11).

**Fig. 4:**
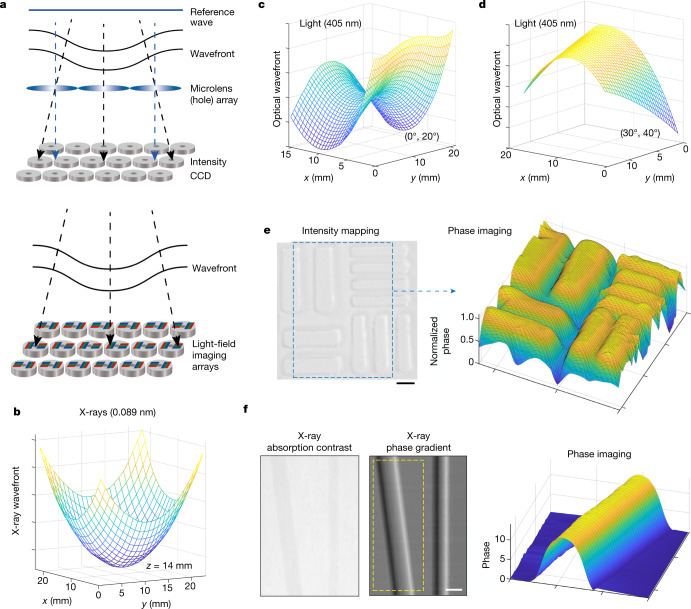
Phase-contrast imaging of X-rays (0.089 nm) and visible light (405 nm) by pixelated colour conversion. **a**, Principles of the Hartmann or Shack–Hartmann wavefront imaging (top) and wavefront imaging based on our 3D light-field sensor arrays (bottom). **b**, Measurement of a diverging wavefront of X-rays 14 mm from the X-ray source. **c**,**d**, Wavefronts measured in the image plane when a lens is illuminated by visible light at (*F*_*x*_ = 0°, *F*_*y*_ = 20°) and (*F*_*x*_ = 30°, *F*_*y*_ = 40°) field angles, respectively. *F*_*x*_ and *F*_*y*_ represent field angles in *x* and *y* directions, respectively. The focal length and aperture of the lens are 60 mm and 25.4 mm, respectively. **e**, Optical intensity image of a patterned PDMS substrate (left) and its phase profile (right) measured with the 3D light-field sensor. The thickness of surface structures is 0.6 mm and the wavelength of the laser is 405 nm. **f**, Absorption contrast image of two commercial PMMA rods (1 mm and 2 mm diameters; 50 kV X-rays) and the corresponding phase-gradient map measured using the 3D light-field sensor. Phase imaging of the yellow dashed-box region is achieved using Supplementary equation (32) of the  Supplementary Information. Scale bar, 1 mm (**e**).

Another important application of pixelated colour conversion is phase-contrast imaging across a broad wavelength range from X-rays to visible light (0.002–550 nm). In phase-contrast imaging with a conventional Shack–Hartmann wavefront sensor, arrays of microlenses record the angle of incidence onto a series of grid points that determine the wavefront (Fig. 4a and Supplementary Information sections 12 and 13). A nanocrystal-array-based light-field sensor can directly measure the specific angle of visible light or X-rays to reconstruct the wavefront without microlens arrays. We first characterized the diverging wavefront of a hard X-ray beam by placing the light-field sensor 14 mm from the X-ray source (Fig. 4b). The measured wavefront curvature agrees well with analytical calculations; the maximum angle measured by the light-field sensor is 40.6°. We also mapped the visible light wavefronts in the image plane when a lens was illuminated by visible light at two different field angles (Fig. 4c,d). Furthermore, phase-contrast imaging was performed using visible light on polydimethylsiloxane (PDMS) patterns and X-rays on commercial polymethylmethacrylate (PMMA) rods (Fig. 4e,f). Surface structures can be seen in greater detail by phase-contrast imaging than by absorption-contrast imaging.

We further compared light-field sensors based on patterned nanocrystal arrays with conventional Shack–Hartmann sensors based on microlens arrays. The fabrication of nanocrystal light-field sensors is highly robust with high uniformity over a large area compared with microlens array fabrication. In our experiment, the spatial sampling density is 400 points per mm^2^, the angular resolution is approximately 0.015° and the dynamic angular range is greater than 80°. By contrast, Shack–Hartmann sensors with the same sampling density typically have a dynamic range of less than 2°. The nanocrystal light-field sensor can be used for a wider spectral range. However, microlens array sensors enable better visible-light collection because of their focusing effect. Compared with the spot localization method with Shack–Hartmann sensors, which is influenced by the quality of the spot and random noise, measuring the colour ratio in nanocrystal light-field sensors can be more robust.

In conclusion, we have presented a pixelated colour conversion strategy based on perovskite nanocrystal arrays for 3D light field detection, absolute spatial positioning, 3D imaging and visible light and X-ray phase-contrast imaging. With its current design, we have achieved a vector sensitivity of around 0.0018° and a wavelength response range of 0.002–550 nm. Although X-ray speckle-tracking methods can achieve an angular resolution close to nanoradians, coherent X-ray sources such as synchrotron radiation are a stringent requirement that cannot easily be met in many experimental and medical applications^18,19^. Further improvement in angular precision is possible by integrating high-end colour detectors. For example, a 30-bit colour display with 10-bit colour depth can yield 1.07 billion possible combinations. With advanced lithography methods and state-of-the-art processing, azimuth detector densities in excess of 10^4^ pixels per mm^2^ might be achieved, which could greatly improve spatial resolution of images. At present, it is not possible to detect the light field beyond 550 nm with high angular resolution using perovskite nanocrystals. However, light-field detection and imaging through pixelated colour conversion can be readily extended to other optical materials. Sn-based perovskite nanocrystals, upconversion nanoparticles that are responsive to the near-infrared light or black phosphorus with tunable bandgaps can expand angular detection to the near-infrared, and even micrometre, wavelength range^40,41^. Furthermore, compared with Shack–Hartmann sensors, light-field sensors based on nanocrystal arrays can be directly integrated into the on-chip optical systems to measure wavefronts or phase. Because azimuth detectors can distinguish only the average vector direction of incident light, not the light from multiple directions like a light-field camera, our light-field sensors measure the average vector direction of light at each pixel. As with light-field cameras, nanocrystal light-field sensors must balance between angular and spatial resolution. Scanning light-field imaging systems can be coupled with nanocrystal arrays to further improve spatial resolution^42^. Nonetheless, the ability to image the wavefront of high-energy X-rays provides powerful solutions for optics testing and beam characterization, which can be used in applications ranging from phase-contrast imaging to gravitational-wave detection.

## Methods

### Chemicals

Caesium carbonate (Cs_2_CO_3_, 99.9%), lead(II) chloride (PbCl_2_, 99.99%), lead(II) bromide (PbBr_2_, 99.99%), lead(II) iodide (PbI_2_, 99.99%), oleylamine (technical grade 70%), oleic acid (technical grade 90%), 1-octadecene (technical grade 90%) and cyclohexane (chromatography grade 99.9%) were purchased from Sigma-Aldrich. A Sylgard 184 silicone elastomer kit was purchased from Dow Corning for the preparation of PDMS substrates. ZnS:Cu^2+^/Mn^2+^ and SrAl_2_O_4_:Eu^2+^/Dy^3+^ phosphors were purchased from Xiucai Chemical.

### Synthesis and characterization

CsPb*X*_3_ (*X* = Cl, Br or I) perovskite nanocrystals were synthesized according to a previously described method^34^. First, caesium oleate was synthesized as a caesium precursor; then CsPb*X*_3_ perovskite nanocrystals were synthesized using the modified hot-injection method (Supplementary Information section 1).

Transmission electron microscopy of the synthesized perovskite nanocrystals was performed using an FEI Tecnai G20 transmission electron microscope with an accelerating voltage of 200 kV. Under visible-light or X-ray excitation, perovskite quantum dots give off narrow and colour-tunable visible emissions. Photoluminescence and radioluminescence spectra were obtained using an Edinburgh FS5 fluorescence spectrophotometer (Edinburgh Instruments) equipped with a miniature X-ray source (Amptek). An advantageous property of perovskites as detectors is their linear response to X-ray dose rate or excitation light power with coverage up to several orders of magnitude. The lowest detectable dose rate for X-ray detection is 10.8 nGy s^−1^ and the lowest detectable power for optical detection is 1 pW mm^−2^. Perovskite quantum dots also exhibit a very fast response (decay time, *τ* = 10.4 ns) to pulsed excitation. These nanocrystals show high photostability under successive or repeated cycles of X-ray irradiation and photoexcitation.

### Principle of the 3D light-field sensor

In spherical coordinates, a detector placed parallel to the *y* axis can measure the angle variation of light around the *y* axis in the XOZ plane (Supplementary Fig. 8). For a detector placed parallel to the *x* axis, it can measure the angle variation of light around the *x* axis in the YOZ plane. Accordingly, for a beam incident from any direction (*θ*, *φ*), detector 1 placed parallel to the *x* axis detects the angle *α*_1_ between the projection of the beam onto the YOZ plane and the *z* axis, whereas detector 2 placed parallel to the *y* axis detects the angle *α*_2_ between the projection of the beam onto the XOZ plane and the *z* axis (Supplementary Fig. 9). The relationships between *α*_1_, *α*_2_ and *θ*, *φ* are as follows:

1 $${\alpha }_{1}=\arctan \left(\frac{y}{z}\right)=\arctan \left(\frac{r\,\sin \,\theta \,\sin \,\phi }{r\,\cos \,\theta }\right)=\,\arctan (\tan \,\theta \,\sin \,\phi )$$

2 $${\alpha }_{2}=\arctan \left(\frac{x}{z}\right)=\arctan \left(\frac{r\,\sin \,\theta \,\cos \,\phi }{r\,\cos \,\theta }\right)=\,\arctan (\tan \,\theta \,\cos \,\phi )$$

where *α*_1_ and *α*_2_ are encoded for the colour output of detectors 1 and 2, respectively. In a specific experiment, *α*_1_ and *α*_2_ are obtained from the CIE tristimulus value of the colour output of detectors 1 and 2, respectively. The *φ* and *θ* values of the beam are then obtained from equations (1) and (2) as follows:

3 $$\theta =\arctan (\sqrt{{\tan }^{2}{\alpha }_{1}+{\tan }^{2}{\alpha }_{2}})$$

4 $$\phi =\arctan \left(\frac{\tan \,{\alpha }_{1}}{\tan \,{\alpha }_{2}}\right)$$

### Fabrication and integration of 3D light-field sensor arrays

The 3D light-field sensor based on pixelated perovskite nanocrystal arrays was fabricated using a simple moulding process (Supplementary Information section 4). First, the pre-patterned Si template was sufficiently washed with heptane. A mixture of SYLGARD silicone elastomer 184 or other silicone elastomer, curing agent and quantum dots or other luminescent materials was prepared. Then, the prepared red-emitting quantum-dot–PDMS ink and blue-emitting quantum-dot–PDMS ink were injected into the corresponding rectangular holes on the Si template and cured at 70–90 °C for 30–60 min. The top of the injected quantum-dot ink must line up with the top of the Si template. Next, 0.5-mm thick PDMS was spun onto the surface of the Si template as an adhesive film. After curing at 70–90 °C for 30 min, the PDMS film patterned with red and blue pixels was moulded off the Si template. Similarly, green-emitting quantum-dot ink was injected into the Si template and cured at 70–90 °C for 30–60 min. Then, a layer of transparent PDMS was coated on the previously processed PDMS film printed with red and blue pixels, which was then overlaid on the green-emitting ink-injected template on the mask aligner holder. Finally, after curing at 70–90 °C for 30–60 min, a film with red, green and blue pixel arrays was obtained by a mould release process. The 3D light-field sensor was formed by integrating the processed pixelated perovskite nanocrystal array film into a colour CCD, with each angle-dependent pixel covering multiple CCD pixels. The colour CCD is a Sony ICX274AL sensor with a chip size of 10 mm × 14 mm (horizontal × vertical), providing 24-bit red, green and blue true colours.

In current 3D printed moulds, the size can be adjusted from tens of micrometres to several millimetres. Large-scale manufacturing is possible through repeated demoulding. This method eliminates the need for complex semiconductor processes and special gases, which greatly reduces processing costs. Fabrication errors typically include random defects and alignment errors (Supplementary Fig. 11 and Supplementary Information section 4). Because the demoulding process used in this work has high machining accuracy and edge defects can be controlled within 0.1%, the random defect error of the entire azimuth detector pixel is almost negligible. A layer-alignment error occurs during processing because of the need to align the top and bottom layers. In cases in which the image distance is much greater than the thickness of a single-colour pixel, alignment deviation will not affect angle measurement.

### Calibration of the 3D light-field sensor

The 3D light-field sensor based on perovskite nanocrystal arrays was calibrated under a collimated light-emitting diode light source. A motorized rotation stage (Daheng Optics, GCD-011060M), a pitch platform and a linear stage were connected to rotate the 3D light-field sensor in θ and ϕ directions. The image sensor was attached to the pitch platform. The pitch platform moves in the θ direction and the rotatory stage moves in the ϕ direction. A linear stage is used to compensate the off-axis movement of the image sensor when it rotates in θ direction. We present selected raw colour images captured during the calibration process to illustrate the working principle of 3D light-field sensor (Supplementary Information section 9). Each panel shows a cropped region of the raw colour image taken at different incident angles. We can observe the angular dependence of the colour output of each pixel. The yellow square represents a vertical angle-sensitive unit and the red square represents a horizontal angle-sensitive unit. When *θ* increases from −40° to 40° with *φ* = 0°, the blueness of the pixel in the yellow square gradually becomes weaker. When *θ* increases from −40° to 40° with *φ* = 90°, the blueness of the pixel in the red square becomes gradually weaker. Once the calibration is completed for the entire range of *θ* and 𝜙, the captured raw images are used as a lookup table for individual angle detection pixels that determine the incident angle of light.

### Three-dimensional imaging procedure

The custom-built optical set-up for 3D imaging consists of a light source and an optical grating to generate multiline structured light on the 3D scene to be imaged (Supplementary Information section 11). The reflected and/or scattered light from the object is collected by a custom-made objective, which consists of two lenses optimized for focal lengths of 100 mm and 25 mm, respectively, enabling maximum angular variation for different object distances identified by the developed high-resolution 3D light-field sensor. In a typical experiment, the parameters of the multiline structured light and the camera as well as the relative distance between them were first calibrated. Next, the colour output of each detector was mapped to the angular arrays according to the calibrated results; then we can calculate the spatial coordinates *x*, *y* and *z* of the object point corresponding to each angular detection unit (Supplementary equations (13)–(17) in the Supplementary Information).

### Spherical X-ray wavefront measurement

A 3D light-field sensor was used to measure the wavefront of spherical hard X-rays (14 keV) (Supplementary Information section 13). The X-ray source produces a divergent beam with a divergence angle of approximately 90° with wavefronts measured at different positions. The farther the 3D light-field sensor is from the X-ray source, the smaller is the measured radius of curvature of the spherical wavefront. Furthermore, from the reconstructed wavefront and slope mapping we can identify the tilt angle between the X-ray source and the sensor (Supplementary Figs. 28 and 29). As a proof of principle, colour data mapping at *z* = 5 mm was used as calibration data for reconstructing wavefronts at other distances *z*, resulting in less-accurate slope measurements with increasing *z*. In practical applications, sampling angles must be obtained in sufficient numbers to ensure angular resolution.

### Phase-contrast imaging procedure

In phase-contrast imaging using collimated UV–visible light, the object is a patterned PDMS substrate with a strip thickness of 0.6 mm. The light-field imaging sensor is placed directly behind the object to capture an image of the changed wavefront. To obtain a nearly collimated beam for X-ray phase-contrast imaging, a copper-column collimator is positioned behind the radiation source. Two commercial PMMA rods of 1 mm and 2 mm diameter are placed behind the radiation source and the light-field imaging sensor detects the changed wavefront. Specifically, the light-field imaging sensor acquires pixelated beam angles, which characterize the phase-gradient distribution. After performing median filtering and integration on the phase gradient, phase mapping can be achieved.

## Online content

Any methods, additional references, Nature Portfolio reporting summaries, source data, extended data, supplementary information, acknowledgements, peer review information; details of author contributions and competing interests; and statements of data and code availability are available at 10.1038/s41586-023-05978-w.

## Supplementary information

Supplementary Information This file contains a table of contents leading and sections 1–14. It includes additional information on material properties, the principle of light-direction detection, device processing, design theory and the experimental apparatus for 3D imaging, system calibration methods, additional imaging data and application demonstrations to support the conclusions of the paper.

## Data Availability

The data that support the findings of this study are available in the Article and its Supplementary Information, and are also available at GitHub (https://github.com/yly1994/color-coded-light-field-imaging.git). Any further data can be obtained from the corresponding author upon reasonable request.

## Code availability

Relevant codes are available at GitHub (https://github.com/yly1994/color-coded-light-field-imaging.git).
