# Supplementary material for: X-ray-to-visible light-field detection through pixelated colour conversion
Source: Nature. 2023 May 10;618(7964):281–6. doi: 10.1038/s41586-023-05978-w (PMC10247359; doi:10.1038/s41586-023-05978-w)
Supplement: Supplementary file 1 — This file contains a table of contents leading and sections 1–14. It includes additional information on material properties, the principle of light-direction detection, device processing, design theory and the experimental apparatus for 3D imaging, system calibration methods, additional imaging data and application demonstrations to support the conclusions of the paper. [file 41586_2023_5978_MOESM1_ESM.pdf]

nature portfolio

https://doi.org/10.1038/s41586-023-05978-w

# Supplementary information

# X-ray-to-visible light-field detection through pixelated colour conversion

In the format provided by the
authors and unedited

# Supplementary Materials for

## X-ray-to-visible light-field detection through pixelated color conversion


Luying Yi[1†], Bo Hou[1†], He Zhao[1,2], Xiaogang Liu[1,2,3,4*]

*[1]Department of Chemistry, National University of Singapore, 117543, Singapore. [2]Joint School of National University of Singapore and Tianjin University, International Campus of Tianjin University, Binhai New City, Fuzhou 350207, China. [3]Center for Functional Materials, National University of Singapore Suzhou Research Institute, Suzhou 215123, China. [4]Institute of Materials Research and Engineering, Agency for Science, Technology and Research, Singapore, 138634, Singapore.*

**Corresponding author. E-mail: chmlx@nus.edu.sg (X. Liu.)*

*[†]These authors contributed equally to this work.*


**Table of Contents**


S1. Synthesis and characterization S2

S2. The positioning principle and error analysis S5

S3. Principle of 3D light-direction detection S8

S4. Fabrication and integration of 3D light-field sensor arrays S12

S5. Geometric model of the 3D imaging system S14

S6. Parameter selection of the 3D imaging system S16

S7. Quantitative analysis of the 3D imaging system. S19

S8. Calibration of the 3D imaging system S22

S9. Calibration of the 3D light-field sensor S24

S10. Factors affecting angular resolution S25

S11. Imaging procedure S27

S12. Wavefront detection principle S31

S13. Spherical X-ray wavefront measurement S33

S14. References S35

## S1. Synthesis and characterization

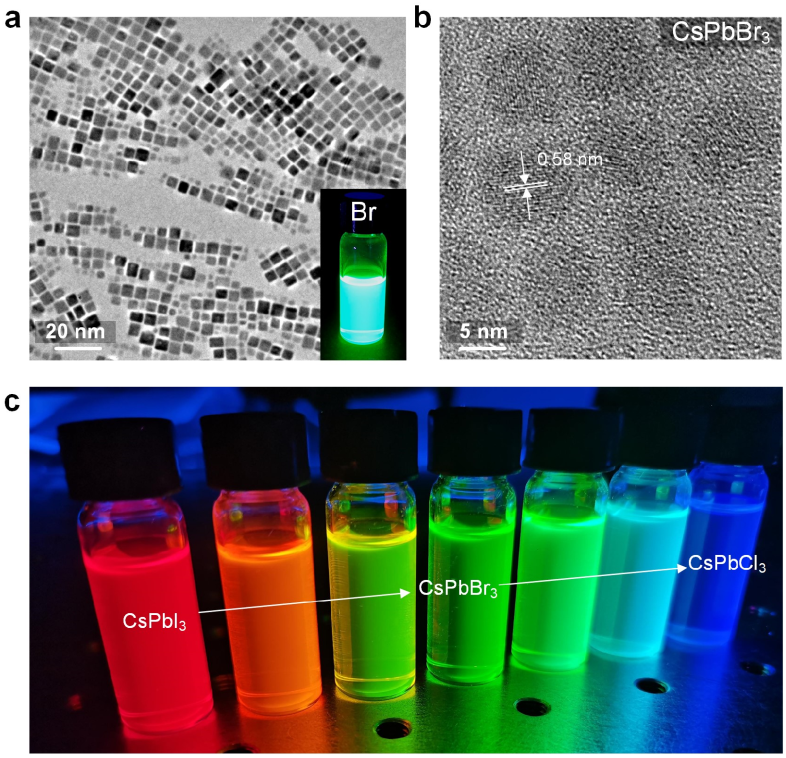


**Supplementary Fig. 1 │ Characterizations of as-synthesized perovskite nanocrystals. a,** Typical TEM image of the cubic perovskite nanocrystals ($CsPbBr_3$). **b,** High-resolution TEM image of the $CsPbBr_3$ nanocrystals. **c,** Optical images of the perovskite nanocrystals dispersed in cyclohexane, recorded under 375-nm ultraviolet excitation.

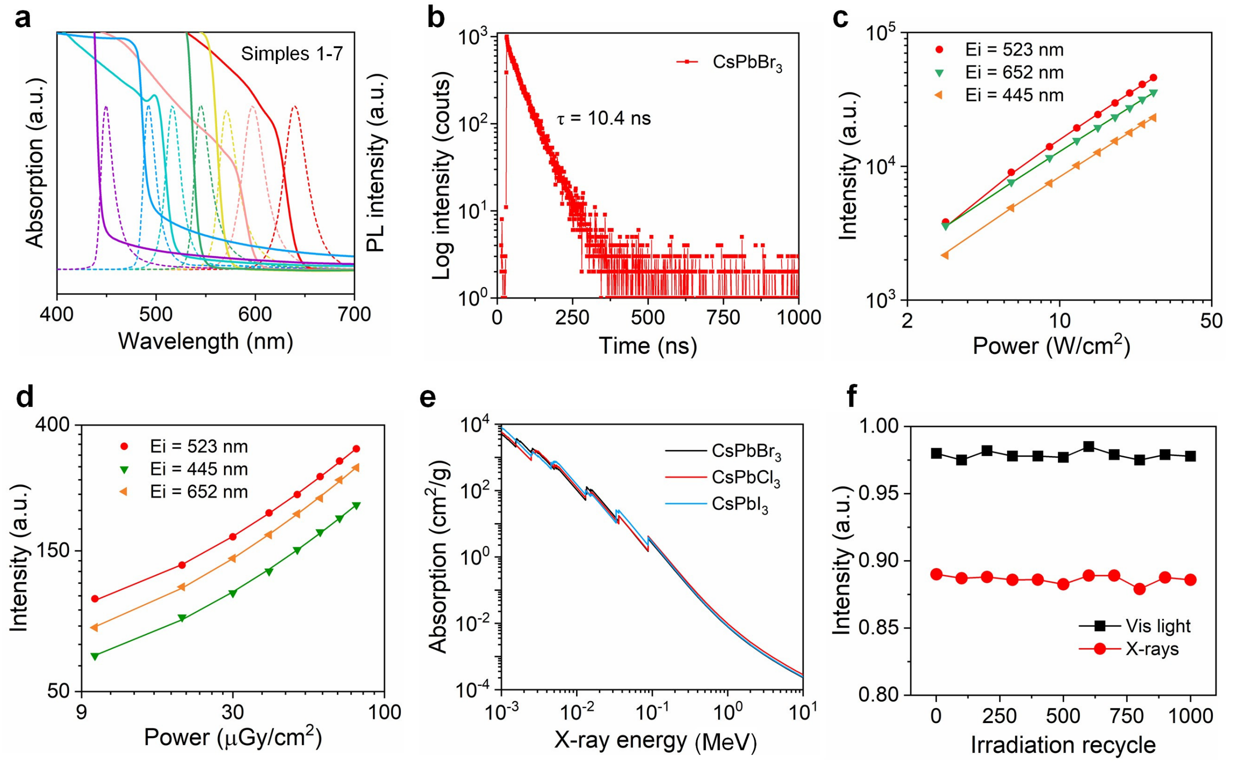


**Supplementary Fig. 2 │ Optical characterization of as-synthesized perovskite nanocrystals. a,** Tunable absorption and luminescence spectra of perovskite nanocrystals as a function of wavelength. **b,** Measured luminescence decay of $CsPbBr_3$ nanocrystals under pulse excitation. The decay time is $\tau = 10.4$ ns. **c,** Luminescence intensity versus the excitation power of visible light (405 nm) for three $CsPbX_3$ perovskite nanocrystals with different emission wavelengths. **d,** Radioluminescence measurements for $CsPbX_3$-based scintillators as a function of dose rate. **e,** Absorption spectra of $CsPbCl_3$, $CsPbBr_3$, and $CsPbI_3$ as a function of X-ray energy. Attenuation coefficients were obtained from ref. [43]. **f,** Photostability of $CsPbBr_3$ nanocrystals under continuous photoexcitation (wavelength $\lambda = 405$ nm; top) and repeated cycles of X-ray excitation at 30 kV with a time interval of 30 s (bottom).

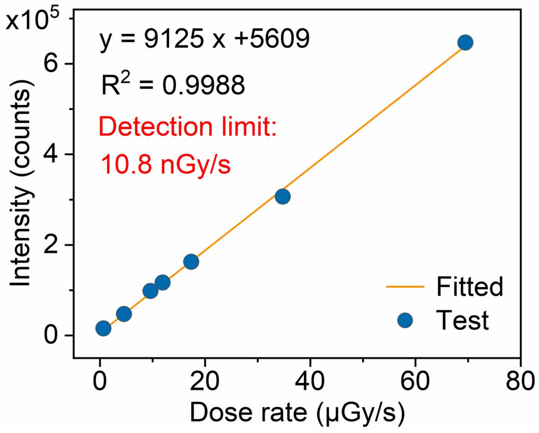


**Supplementary Fig. 3 │ Measurement of the detection limit for perovskite nanocrystal films irradiated with an X-ray source**. $CsPbBr_3$ nanocrystals were used for X-ray detection by converting high-energy photons into visible emission that can be readily detected by a photomultiplier tube. The detection limit of 10.8 nGy $s^{-1}$ is determined by the slope of the fitting line with a signal-to-noise ratio of 3.

## S2. The positioning principle and error analysis

The photoluminescence part of the azimuth detector consists of three sets of $CsPbX_3$ nanocrystals, which emit red, green, and blue light. Since the absorption of light or radiation of each part changes with the incident direction of light, there is a mapping between the color of luminescence and the azimuth angle of excitation light. Each azimuth detector can determine the angle $\alpha$ of the incident beam with respect to the reference plane, so three such azimuth detectors can be arranged to locate the spatial position of the excitation source (**Supplementary Fig. 4**). In the three-dimensional Cartesian coordinate system, detector A and detector B are perpendicular to the XOY plane at coordinates ($b$, 0, 0) and coordinates (0, 0, 0), and cylinder C is arranged parallel to the XOY plane along the Y axis. Assuming that the X axis is the reference direction, the projection of the light or radiation source $S$ onto the XOY plane is $S'$, the angle between the line (connecting $S'$ and detector A) and the reference direction is $\theta_1$, and the angle between the line (connecting $S'$ and detector B) and the reference direction is $\theta_2$. The angle between the line (connecting $S$ and the detector C) and the XOY plane is $\theta_3$. $\theta_1$, $\theta_2$, and $\theta_3$ are determined by the color of the luminescence of azimuth detectors A, B and C, respectively. Therefore, the spatial position ($x$, $y$, $z$) of the source $S$ can be solved by the following formula:

$$\begin{cases} \tan\theta_1 = \dfrac{y}{x-b} \\ \tan\theta_2 = \dfrac{y}{x} \\ \tan\theta_3 = \dfrac{z}{x} \end{cases} \Rightarrow \begin{cases} x = \dfrac{b\tan\theta_1}{\tan\theta_1 - \tan\theta_2} \\ y = \dfrac{b\tan\theta_1\tan\theta_2}{\tan\theta_1 - \tan\theta_2} \\ z = \dfrac{b\tan\theta_1\tan\theta_3}{\tan\theta_1 - \tan\theta_2} \end{cases} \tag{1}$$

The positioning errors d$x$, d$y$ and d$z$ of the source $S$ depend on the angular detection error d$\theta$ of each azimuth detector, the distance $b$, and the position coordinates $x$, $y$, and $z$ of the source. The d$x$, d$y$ and d$z$ as a function of d$\theta$ are:

$$dx = b\frac{(\tan\theta_1)'d\theta}{\tan\theta_1 - \tan\theta_2} + b\tan\theta_1\left[\frac{(\tan\theta_1)'d\theta - (\tan\theta_2)'d\theta}{-(\tan\theta_1 - \tan\theta_2)^2}\right] \tag{2}$$

$$= b\left[\frac{\tan\theta_1(\tan\theta_2)' - \tan\theta_2(\tan\theta_1)'}{(\tan\theta_1 - \tan\theta_2)^2}\right]d\theta = b\left[\frac{\sin\theta_1\cos\theta_1 - \sin\theta_2\cos\theta_2}{\left(\sin\theta_1\cos\theta_2 - \sin\theta_2\cos\theta_1\right)^2}\right]d\theta$$

$$= b\left[\frac{\cos(\theta_1+\theta_2)}{\sin(\theta_1-\theta_2)}\right]d\theta,$$

$$dy = b\frac{\tan\theta_2(\tan\theta_1)'d\theta + \tan\theta_1(\tan\theta_2)'d\theta}{\tan\theta_1 - \tan\theta_2} + b\tan\theta_1\tan\theta_2\left[\frac{(\tan\theta_1)'d\theta - (\tan\theta_2)'d\theta}{-(\tan\theta_1 - \tan\theta_2)^2}\right]$$

$$= b\left[\frac{\tan^2\theta_1(\tan\theta_2)' - \tan^2\theta_2(\tan\theta_1)'}{(\tan\theta_1 - \tan\theta_2)^2}\right]d\theta = b\left[\frac{\sin^2\theta_1 - \sin^2\theta_2}{(\sin\theta_1\cos\theta_2 - \sin\theta_2\cos\theta_1)^2}\right]d\theta \quad (3)$$

$$= b\left[\frac{\sin(\theta_1 + \theta_2)}{\sin(\theta_1 - \theta_2)}\right]d\theta,$$

and

$$dz = dx\tan\theta_3 + x\sec^2\theta_3 d\theta \quad (4)$$

Theoretical analysis shows that d$x$, d$y$, and d$z$ are all positively correlated with d$\theta$, d$x$ is positively correlated with $b$, and d$y$ and d$z$ are negatively correlated with $b$. Positioning errors are closely related to position of $S$ (**Supplementary Fig. 5**). As a proof-of-concept, we fabricated three azimuth detectors arranged according to the schematic diagram in Supplementary Fig. 4b. We achieved 3D spatial localization of the X-ray source with a localization accuracy of approximately 0.5% (**Supplementary Fig. 6**).

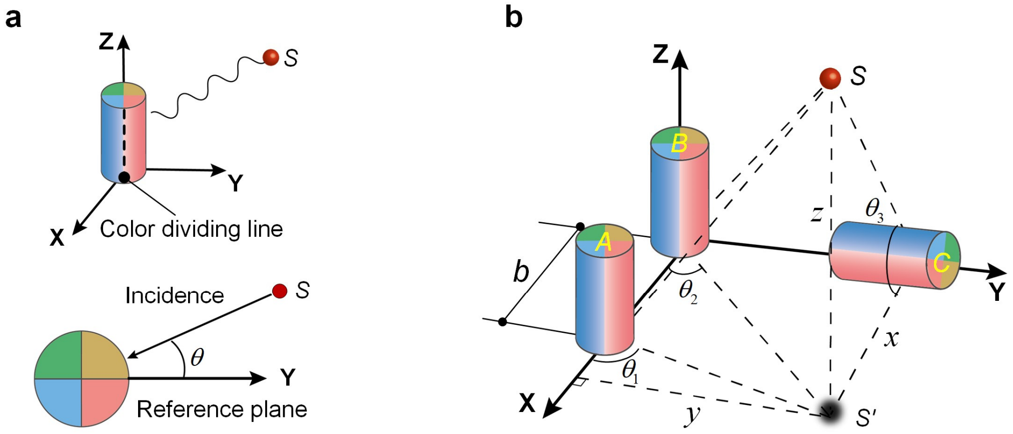


**Supplementary Fig. 4 | Schematic of an azimuth detection scheme and a source positioning scheme**. **a,** Schematic diagram of a single azimuth detector. With a color dividing line as the reference direction, the detector can identify the angle $\alpha$ between the incident beam and the reference direction. **b,** Schematic diagram of the source localization principle. The spatial position ($x$, $y$, $z$) of the source $S$ can be solved from $\theta_1$, $\theta_2$, and $\theta_3$, which are determined by the color of the luminescence of detectors A, B and C, respectively.

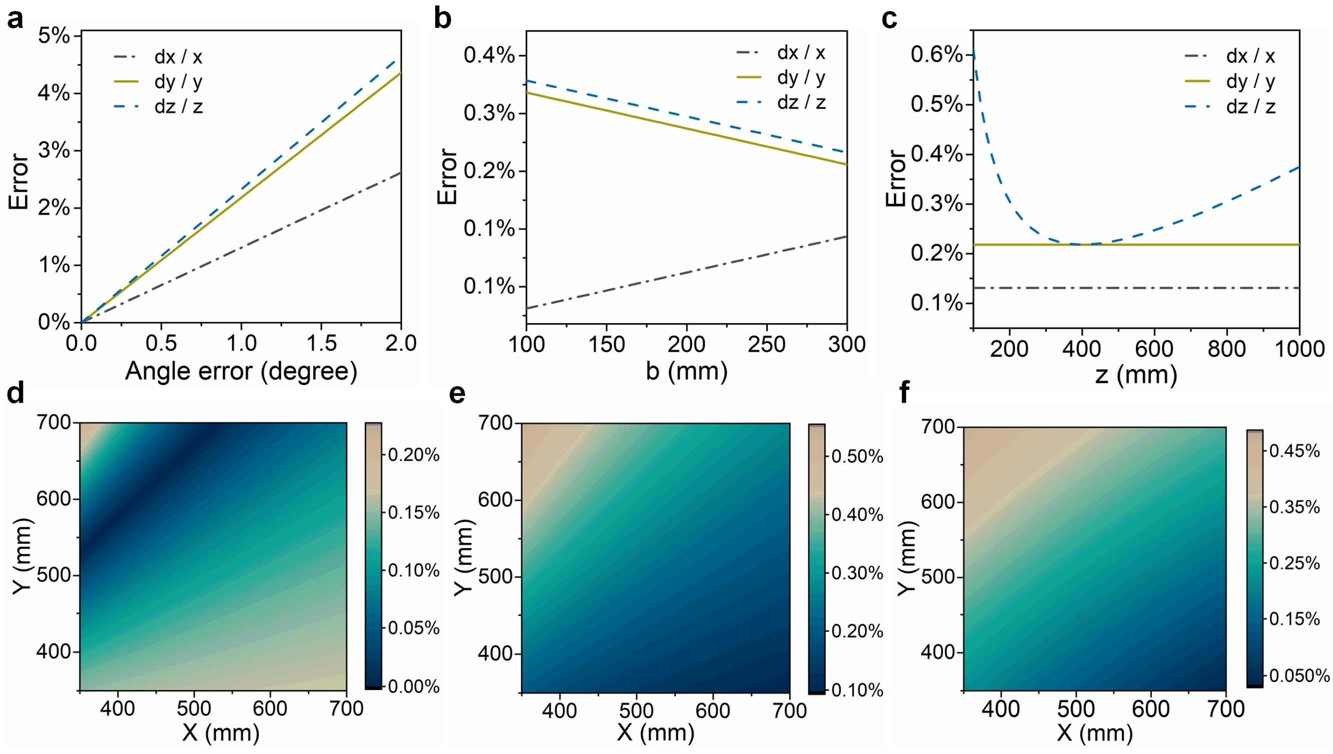


**Supplementary Fig. 5 │ Theoretical analysis of the positioning errors**. **a,** d$x$, d$y$ and d$z$ as a function of angular detection error d$\theta$. **b,** d$x$, d$y$ and d$z$ versus $b$. **c,** The relationship between the positioning errors and the coordinate $z$ of the excitation source $S$. **d-f**, Positioning errors d$x$ (d), d$y$ (e), and d$z$ (f) as a function of the spatial coordinates $x$ and $y$ of the excitation source.

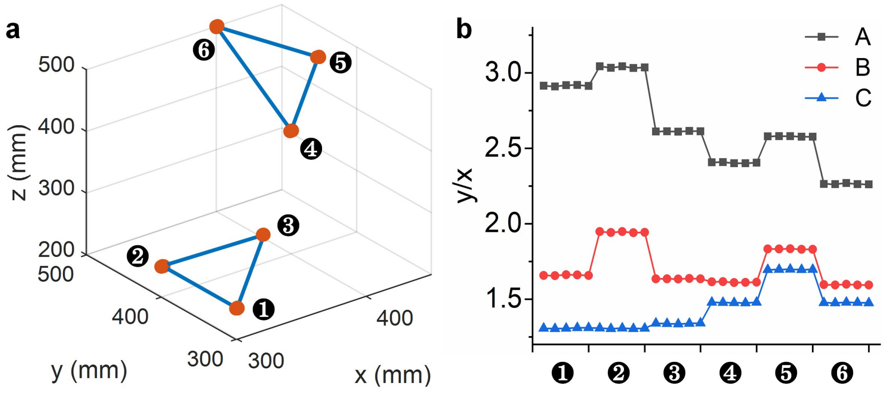


**Supplementary Fig. 6 │ Experimental demonstration of 3D Positioning**. **a**, Results of locating the ray source using the three azimuth detectors. **b**, Chromaticity responses of the three azimuth detectors (A, B, and C) corresponding to the six positions in panel **a**.

## S3. Principle of 3D light-direction detection

We first define that azimuth is the counterclockwise angle measured from the positive direction of the x-axis on the x-y plane, and elevation is the elevation angle formed with the x-y plane (**Supplementary Fig. 7a**). The photoluminescence part of the azimuth detector consists of three sets of $CsPbX_3$ nanocrystals that emit red, green, and blue light. Since the absorption of light or radiation of each part changes with the incident direction of the light, there is a mapping between the color of luminescence and the azimuth angle of the excitation light. In Fig. S7a, the color change caused by the change of the elevation angle of the light is not obvious (its effect on the color output can be eliminated during calibration), but the change of the azimuth angle can cause a very large color change. Therefore, we give an approximate solution for the color function of the light output from a single detector when the incident ray has an elevation angle of 0° but different azimuth angles. In this case, it is more convenient to analyze the color as a function of azimuth $\alpha$ using the top view of the detector (**Supplementary Fig. 7b**). The white circle with a radius of $r$ in the center represents the transparent material used to transmit light to the bottom of the detector. When light is incident from the direction shown in the figure, materials closer to the incident direction emit stronger light due to the exponential decay of the excitation light. As long as the power of the light to be measured is not too low, the color response does not depend on the light power because the light intensity does not affect the light color.

For simplicity, we assume $r = 0$ and assume that the luminescence of the materials near the incident direction is uniform, while the luminescence of the materials far from the incident direction is neglected. This approximation affects the final chromaticity response function, but can still show the huge color change due to the change of azimuth angle $\alpha$. Under this approximation, the output light spectrum of the nanocrystal sensor can be expressed as:

$$\mathrm{S}(\lambda) = (\pi R^2 \frac{\theta_r(\alpha)}{2\pi})\eta_r(\lambda) + (\pi R^2 \frac{\theta_g(\alpha)}{2\pi})\eta_g(\lambda) + (\pi R^2 \frac{\theta_b(\alpha)}{2\pi})\eta_b(\lambda) \tag{5}$$

where $\eta_r(\lambda)$, $\eta_g(\lambda)$ and $\eta_b(\lambda)$ are the luminescence spectra of the red, green and blue luminescent materials, respectively. $\theta_r(\alpha)$, $\theta_g(\alpha)$, and $\theta_b(\alpha)$ represent the angle of the area at which the three materials are irradiated. They are functions of azimuth $\alpha$ and can be expressed as a piecewise function:

$$\theta_r = \begin{cases} \frac{2\pi}{3} - \alpha, & \alpha \in [0, \frac{2\pi}{3}) \\ \alpha - \frac{2\pi}{3}, & \alpha \in [\frac{2\pi}{3}, \frac{4\pi}{3}) \\ \frac{2\pi}{3}, & \alpha \in [\frac{4\pi}{3}, 2\pi) \end{cases} ; \theta_g = \begin{cases} \frac{2\pi}{3}, & \alpha \in [0, \frac{2\pi}{3}) \\ \frac{4\pi}{3} - \alpha, & \alpha \in [\frac{2\pi}{3}, \frac{4\pi}{3}) \\ \alpha - \frac{4\pi}{3}, & \alpha \in [\frac{4\pi}{3}, 2\pi) \end{cases} ; \theta_b = \begin{cases} \alpha, & \alpha \in [0, \frac{2\pi}{3}) \\ \frac{2\pi}{3}, & \alpha \in [\frac{2\pi}{3}, \frac{4\pi}{3}) \\ 2\pi - \alpha, & \alpha \in [\frac{4\pi}{3}, 2\pi) \end{cases} \tag{6}$$

Substituting Eq. (6) into Eq. (5), we obtain the functional relationship between the output spectrum S(λ) of the detector and the azimuth *α* of the measured light. The spectrum S(λ) can be converted into CIE color tristimulus values (*X*, *Y*, and *Z*) by the following formula:

$$\begin{aligned} X &= K \cdot \sum_{\lambda}[CIE1931Std\_X(\lambda)\cdot S(\lambda)] \\ Y &= K \cdot \sum_{\lambda}[CIE1931Std\_Y(\lambda)\cdot S(\lambda)] \\ Z &= K \cdot \sum_{\lambda}[CIE1931Std\_Z(\lambda)\cdot S(\lambda)] \end{aligned} \tag{7}$$

Where *K* is the proportional coefficient, and *CIE1931Std* is the standard data.

The relative coefficients *x* and *y* of the three primary colors can be obtained by:

$$\begin{aligned} x &= X/(X+Y+Z) \\ y &= Y/(X+Y+Z) \end{aligned} \tag{8}$$

Then the color corresponding to each azimuth *α* can be intuitively displayed in the chromaticity diagram. In our experiment, the chromaticity response of the output light shows a large triangle on the chromaticity diagram when the azimuth *α* varies from 0 to 360 degrees.

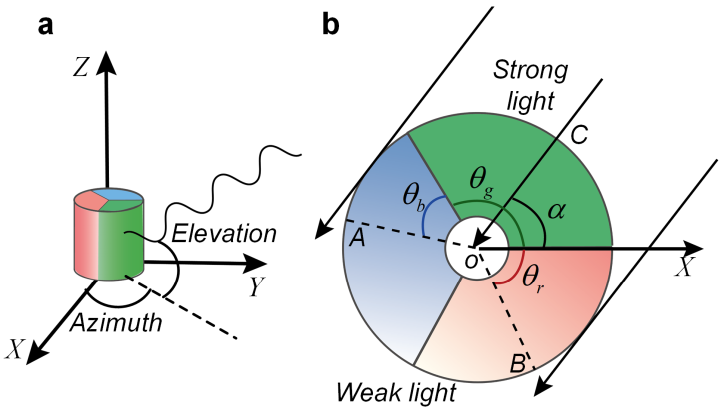


**Supplementary Fig. 7 │ Analysis of the principle of angle-to-color conversion. a,** Schematic diagram of a single detector. Azimuth is the counterclockwise angle measured from the positive direction of the x-axis on the x-y plane, and elevation is the elevation angle formed with the x-y plane. **b**, Top view of the single detector shown in **a**. The detector being analyzed consists of three types of materials, each emitting red, green, and blue colored light. The materials closer to the incident direction emit stronger light due to the exponential decay of the excitation light. The white circle with a radius of *r* in the middle represents the transparent material used to transmit the light to the bottom of the detector.

Two azimuth detectors arranged perpendicular to each other can perform 3D omnidirectional light-field detection. In spherical coordinates, a detector placed parallel to the y-axis (Supplementary Fig. 8a) can measure

the angle variation of light around the y-axis in the XOZ plane. That is, when the light drawn in blue scans along the direction indicated by the red arrow, changes in the angle $\alpha_2$ can be detected. For a detector that is parallel to the x-axis (Supplementary Fig. 8b), it can measure the angle variation of the light around the x-axis in the YOZ plane. That is, when the light drawn in blue scans along the direction indicated by the red arrow, changes in the angle $\alpha_1$ can be detected. Accordingly, in spherical coordinates (Supplementary Fig. 9a), for a beam incident from any direction ($\theta$, $\varphi$), detector 1 detects the angle $\alpha_1$ between the projection of the beam onto the YOZ plane and the $Z$ axis, while detector 2 detects the angle $\alpha_2$ between the projection of the beam onto the XOZ plane and the $Z$ axis. The relationships between $\alpha_1$, $\alpha_2$ and $\theta$, $\varphi$ are as follows:

$$\begin{aligned}\alpha_1 &= \arctan(\frac{y}{z}) = \arctan(\frac{r\sin\theta\sin\varphi}{r\cos\theta}) \\ &= \arctan(\tan\theta\sin\varphi)\end{aligned} \tag{9}$$

$$\begin{aligned}\alpha_2 &= \arctan(\frac{x}{z}) = \arctan(\frac{r\sin\theta\cos\varphi}{r\cos\theta}) \\ &= \arctan(\tan\theta\cos\varphi)\end{aligned} \tag{10}$$

where $\alpha_1$ and $\alpha_2$ are encoded for the color output of detectors 1 and 2, respectively. In a specific experiment, $\alpha_1$ and $\alpha_2$ are obtained from the CIE tristimulus value of the color output of detectors 1 and 2, respectively. The azimuth angle $\varphi$ and elevation angle $\theta$ of the beam are then obtained from the following expressions derived from equations (9) and (10):

$$\theta = \arctan(\sqrt{\tan^2\alpha_1 + \tan^2\alpha_2}) \tag{11}$$

$$\varphi = \arctan(\frac{\tan\alpha_1}{\tan\alpha_2}) \tag{12}$$

We further designed a 3D light-direction image array using perovskite nanocrystals in which adjacent pixels are perpendicular to each other. For simplicity, the angle detected by detectors parallel to the $x$-axis is denoted by $\alpha_{i,j}$ ($i$ and $j$ refer to the rows and columns of the nanocrystal arrays), and the angle detected by detectors parallel to the $y$-axis is denoted by $\beta_{i,j}$. Each of the two azimuth detectors, which are perpendicular to each other, can reconstruct the angle of the beam incident at the center of the two pixels. For example, $\alpha_{1,1}$ and $\beta_{1,2}$ can be used to calculate the 3D angle of the beam incident at point $s_{11}$, whereas $\beta_{2,1}$ and $\alpha_{1,1}$ can be used to calculate the 3D angle of the beam incident at point $s_{21}$.

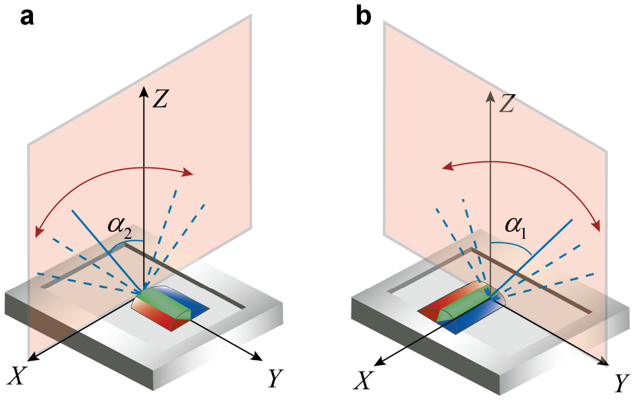


**Supplementary Fig. 8 │Configuration and principle of angle measurement of nanocrystal light-field sensors. a**, A detector placed parallel to the y-axis. When the light drawn in blue scans along the direction indicated by the red arrow, the changes in the angle $\alpha_2$ can be detected. **b**, A detector placed parallel to the x-axis. When the light drawn in blue scans along the direction indicated by the red arrow, the changes in the angle $\alpha_1$ can be detected.

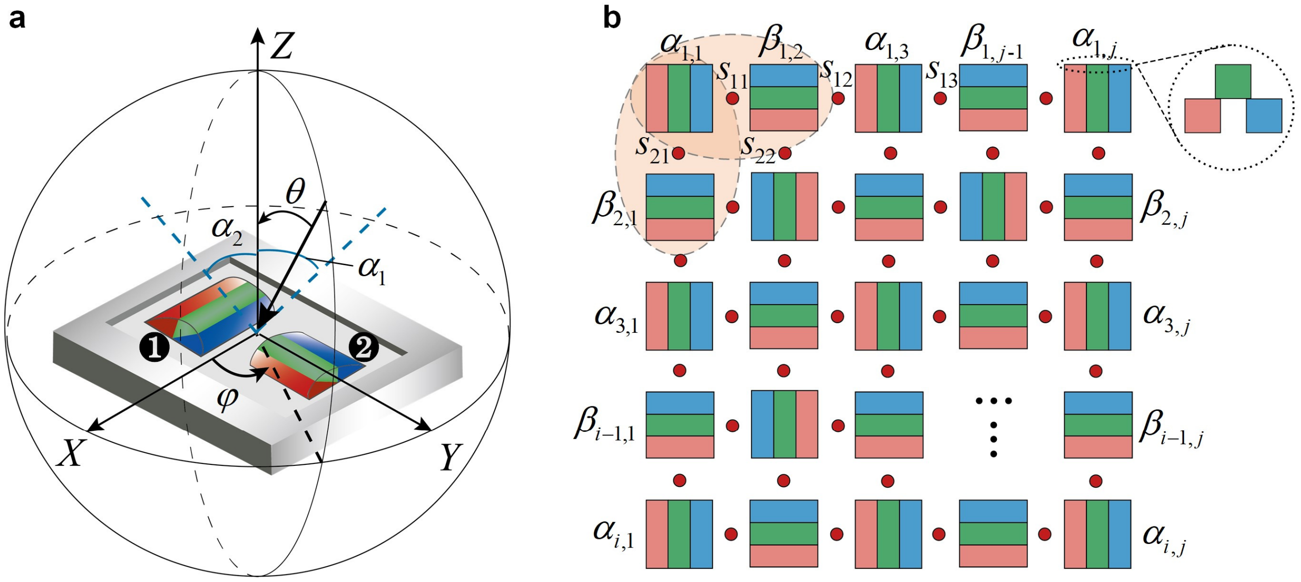


**Supplementary Fig. 9 │ Schematic diagram of 3D light-field detection and detector pixel arrangement**. **a,** Schematic depiction of the relationship between the angle ($\theta$, $\varphi$) of the incident beam in the spherical coordinate system and the angles $\alpha_1$ and $\alpha_2$ resolved for the colors of the two azimuth detectors that are perpendicular to each other. **b,** Top view of the pixel arrangement of 3D light-field sensor arrays. The two detectors circled by the yellow ellipse can reconstruct the angle of the beam incident on the center point of the ellipse. The inset depicts the side view of a patterned pixel.

## S4. Fabrication and integration of 3D light-field sensor arrays

*Processing steps for the light-field detector array*

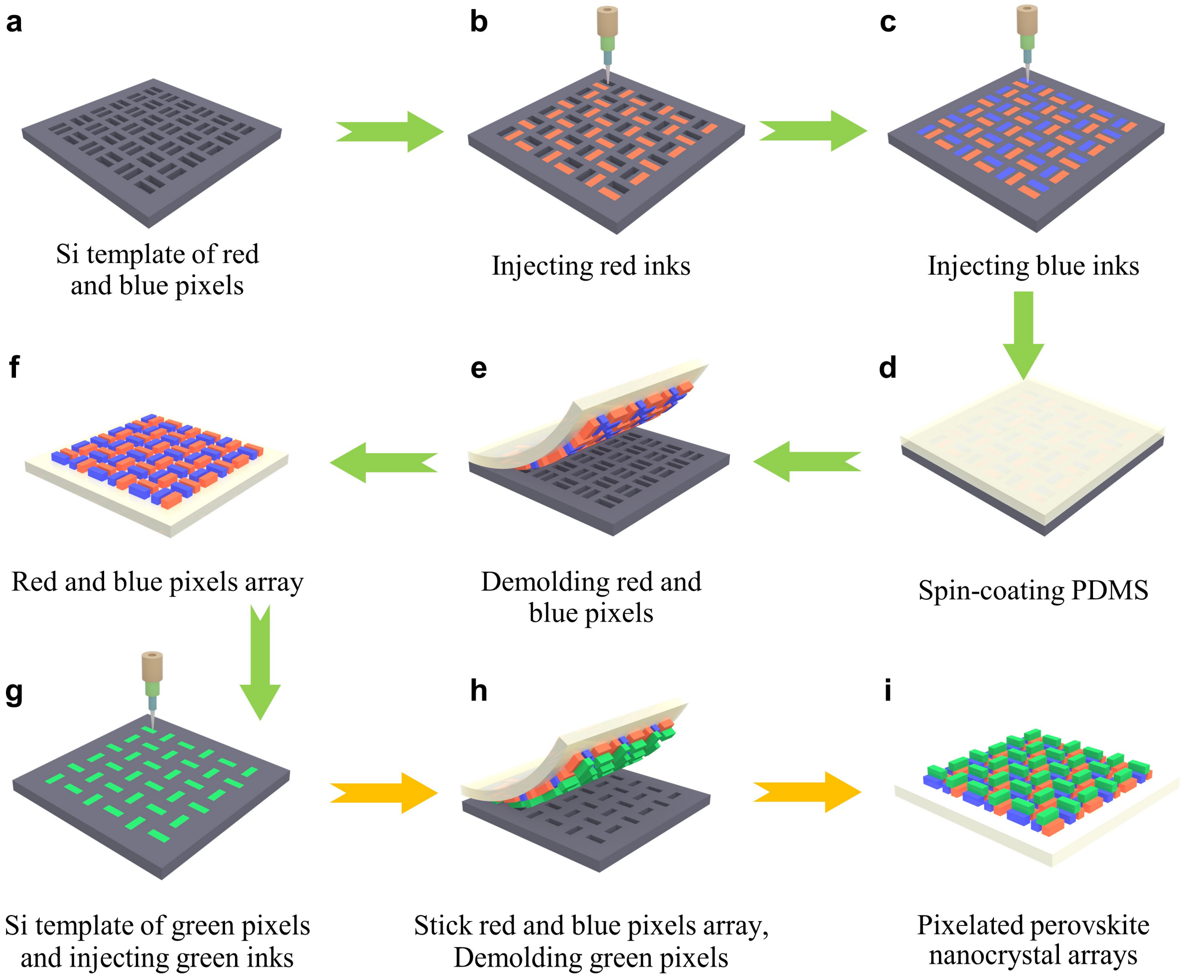


**Supplementary Fig. 10 │ Schematic of the fabrication process of the pixelated perovskite nanocrystal arrays. a,** Pre-patterned Si templates used to fabricate red and blue pixel arrays. **b,** The prepared red-emitting QD-PDMS ink is injected into the corresponding rectangular holes of the template using a direct printing method. **c,** The prepared blue-emitting QD-PDMS ink is injected into the corresponding rectangular holes. **d,** The PDMS is then spun onto the blue and red ink printed template and then heat-treated in a vacuum oven for 30 minutes. **e,** The film printed with the red and blue pixel arrays was obtained by demolding. **f,** The gaps between red and blue pixels were filled with transparent PDMS. **g,** The prepared green QD-PDMS ink was injected into the rectangular holes of the prefabricated template. **h,** The processed PDMS film with red and blue pixel arrays from step F was overlaid on the green ink printed Si template and heat-treated in a vacuum oven for 30 minutes. **i,** The film printed with red, green and blue pixel arrays was obtained by demolding.

*Error analysis of the fabrication and calibration*

A highly robust flat processing is used to fabricate azimuth detector arrays. Typical fabrication errors include random defects and misalignment (**Supplementary Fig. 11**). The demolding process used in this work has high processing accuracy and edge defects can be controlled within 0.1%. The random defect error of the entire azimuth detector pixel is almost negligible due to the averaging effect. Because alignment is required between the upper and lower material layers, an alignment error exists as shown in Supplementary Fig. 9. In the figure, $w$ represents the thickness of a single-color pixel, $D$ represents the vertical distance between the measured object and detector, $\Delta d$ denotes the alignment deviation (<2%), and $\Delta\theta_1$ denotes the angle measurement deviation, which is proportional to the measurement distance. When $w \ll D$, the angle deviation can be ignored.

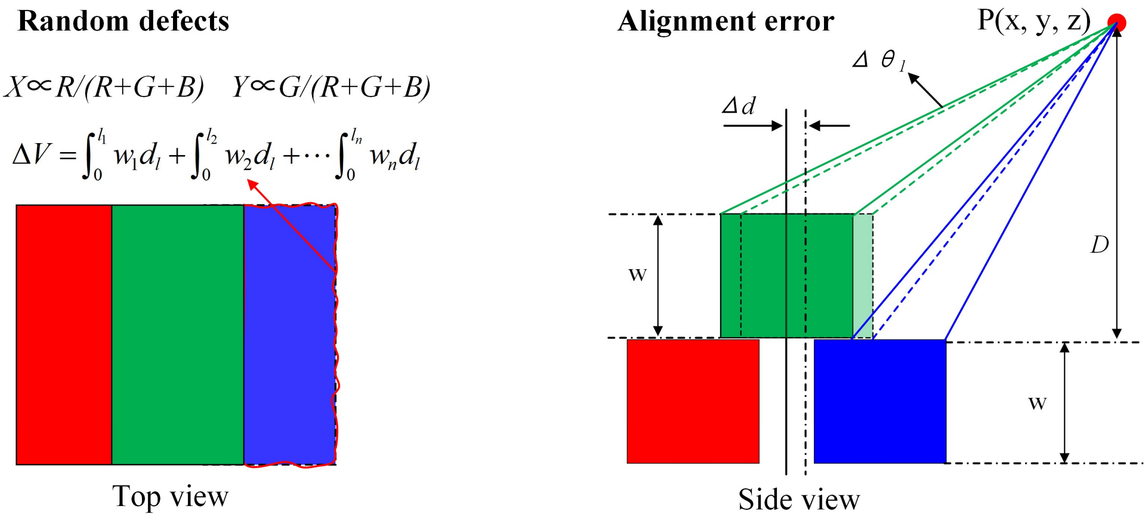


**Supplementary Fig. 11 | Typical fabrication errors caused by random defects and misalignment.**

## S5. Geometric model of the 3D imaging system

The 3D imaging scheme used was the triangulation method based on multiline structured light illumination. For simplicity, we first analyzed the situation under single-line structured light illumination (**Supplementary Fig. 12**).

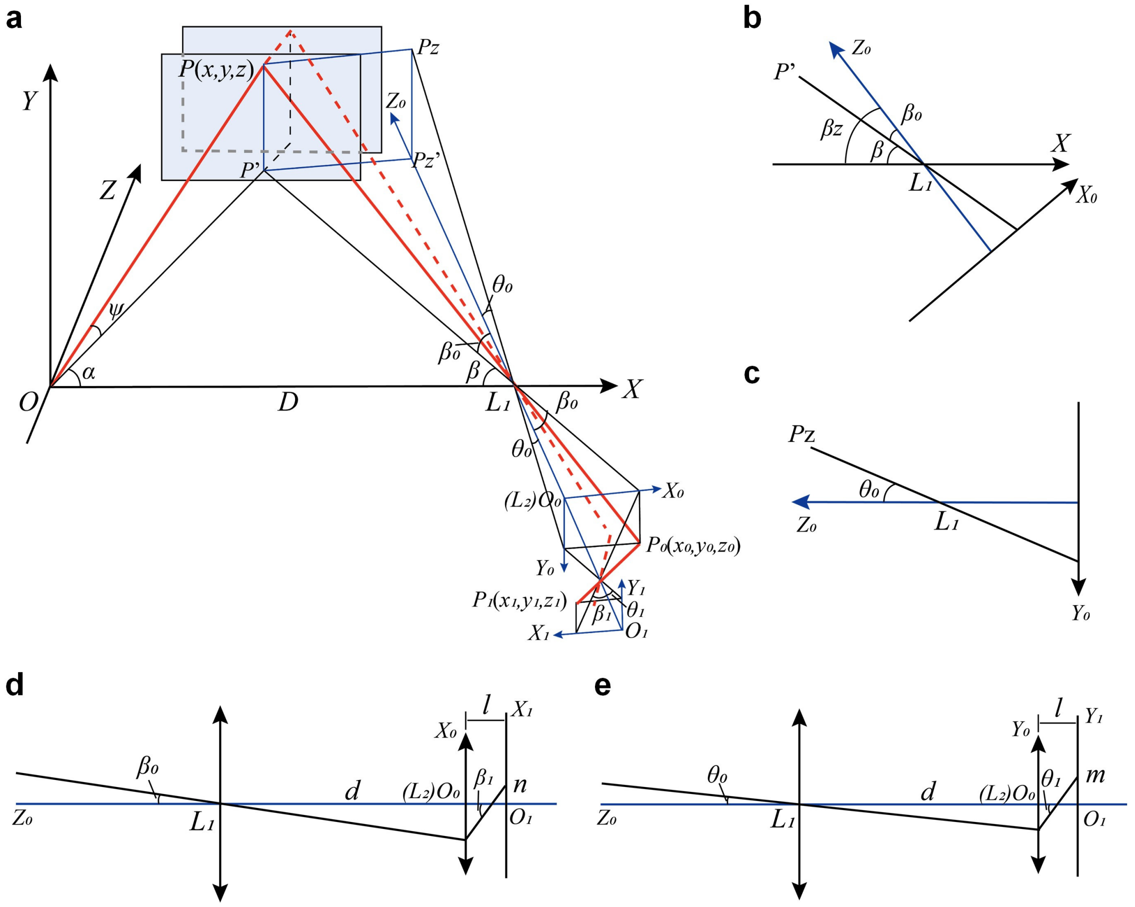


**Supplementary Fig. 12 │ Geometric model of the 3D imaging system based on the triangulation method. a,** Geometric model of the imaging system used in the experiments. The light beam *OP* emitted from the light source irradiates the object point *P*. The light beam $PL_1$ reflected by the object is collected and refracted onto the angle detector plane $X_1O_1Y_1$ through lens 1 and lens 2. **b,** Projections of the coordinate system of lens 1 onto the XOZ plane. **c,** Projections of the coordinate system of the lens 1 onto the $Y_0O_0Z_0$ plane. **d,** Projections of the camera's coordinate system onto the XOZ plane. **e,** Projections of the camera's coordinate system onto the $Y_0O_0Z_0$ plane.

To increase the change of the incident angle on the detector with distance changes and reduce the lateral movement of the light spot on the detector, we designed an objective composed of two lenses. In

Supplementary Fig. 12, $L_1$ and $L_2$ are the center points of lens 1 and lens 2, respectively. The light source at point $O$ emits a single-line structured light perpendicular to the XOZ plane, and the distance between the points $O$ and $L_1$ is $D$. For each object point $P$ ($x$, $y$, $z$) irradiated by the single-line structured light, its image by lens 1 in the $X_0Y_0Z_0O_0$ coordinate system of lens 2 is located at point $P_0$ in the $X_0O_0Y_0$ plane. The $X_0O_0Z_0$ plane and the XOZ plane are coplanar. The angle between the ray $OP$ and the XOZ plane is $\varphi$, and the angle between the projection $OP$' of ray $OP$ onto the XOZ plane and the OX axis is $\alpha$. The angle between the optical axis $O_0Z_0$ of lens 1 and $OL_1$ is $\beta_z$. The angle between the projection $P'L_1$ of ray $PL_1$ onto the $X_0O_0Z_0$ plane and the optical axis $O_0Z_0$ is $\beta_0$, and the angle between the projection $P_zL_1$ of the ray $PL_1$ onto the $Y_0O_0Z_0$ plane and the optical axis $O_0Z_0$ is $\theta_0$. The projections of coordinate system of the lens 1 onto the XOZ plane and the $Y_0O_0Z_0$ plane are shown in Supplementary Fig. 12b and 12c. The light $L_1P_0$ is refracted by lens 2 onto the detector plane $X_1O_1Y_1$. The projection of the camera's coordinate system onto the XOZ plane and the $Y_0O_0Z_0$ plane are shown in Supplementary Fig. 12d and 12e, respectively. The angle between the projection of ray $P_0P_1$ onto the $X_1O_1Z_1$ plane and the optical axis $O_0Z_0$ is $\beta_1$, and the angle between the projection of the ray $P_0P_1$ onto the $Y_1O_1Z_1$ plane and the optical axis $O_1Z_0$ is $\theta_1$. The distance between lens 1 and lens 2 is $d$, and the distance between lens 2 and the detector plane is $l$. $n$ and $m$ represent the number of pixels on the detector in the X and Y directions, respectively.

According to the geometric relations in Supplementary Fig. 12, the position coordinates $x$, $y$, $z$ of the object point $P$ can be solved:

$$z = \frac{D}{\cot\alpha + \cot(\beta_z - \beta_0)} \tag{13}$$

$$x = z\cot\alpha \tag{14}$$

$$y = \frac{z}{\sin(\beta_z - \beta_0)}\cos\beta_0 \tan\theta_0 \tag{15}$$

$$\tan\beta_0 = (l\tan\beta_1 - ns)/d \tag{16}$$

$$\tan\theta_0 = (l\tan\theta_1 - ms_1)/d \tag{17}$$

where $s$ and $s_1$ represent the dimensions of a single pixel of the detector in the X and Y directions, respectively.

In a specific experiment, $\alpha$, $\beta_z$, $D$, $d$, and $l$ need to be calibrated in advance. $\beta_1$ and $\theta_1$ are obtained from the color output of angle detection, and then the coordinates $x$, $y$, $z$ of object $P$ are solved by formula (13)–(17).

## S6. Parameter selection of the 3D imaging system.

In the 2D scheme of the designed imaging system in **Supplementary Fig. 13**, at a certain distance $z$, the lateral position $x$ and the angle $\beta_t$ between the reflected or scattered light ray $PL_1$ and the $X$ axis are:

$$x = z\cot(\alpha) \tag{18}$$

and

$$\beta_t = \arctan[z/(D-x)] \tag{19}$$

According to the geometric relationship in Supplementary Fig. 13 and the Gaussian formula in geometric optics, the object distance $l_1$ and the image distance $l_1$' of lens 1 are:

$$l_1 = -\frac{z}{\sin\beta_t}\cos(\beta_t - \beta_z) \tag{20}$$

and

$$\frac{1}{l_1'} - \frac{1}{l_1} = \frac{1}{f_1} \quad \Rightarrow \quad l_1' = \frac{f_1 l_1}{l_1 + f_1} \tag{21}$$

where $f_1$ is the focal length of lens 1 and $\beta_z$ is the angle between the optical axis of lens 1 and the coordinate axis OX.

The object distance $l_2$ and the image distance $l_2$' of lens 2 are:

$$l_2 = l_1' - d \text{, and } l_2' = \frac{f_2 l_2}{l_2 + f_2} \tag{22}$$

where $f_2$ is the focal length of lens 2.

The vertical magnifications of lens 1 ($\beta_1$), lens 2 ($\beta_2$), and the combined system ($\beta$) are:

$$\beta_1 = \frac{l_1'}{l_1} = \frac{f_1}{l_1 + f_1}, \beta_2 = \frac{f_2}{l_2 + f_2}, \text{ and } \beta = \beta_1\beta_2 = \frac{f_1 f_2}{f_1 l_1 - (l_1 + f_1)(d - f_2)} \tag{23}$$

Therefore, the height of images on the primary imaging plane and the detector imaging plane are:

$$y_1' = y_1\beta_1, \text{ and } y_2' = y_1\beta \tag{24}$$

where $y_1 = -l_1 \tan(\beta_t - \beta_z)$.

The angle between the light beam incident onto the primary image plane and the optical axis is:

$$\beta_0 = \beta_t - \beta_z \tag{25}$$

The angle between the light beam incident onto the detector imaging plane and the optical axis is:

$$\beta_0' = \arctan\left[\frac{d\tan(\beta_0) + y_2'}{l_2'}\right] \tag{26}$$

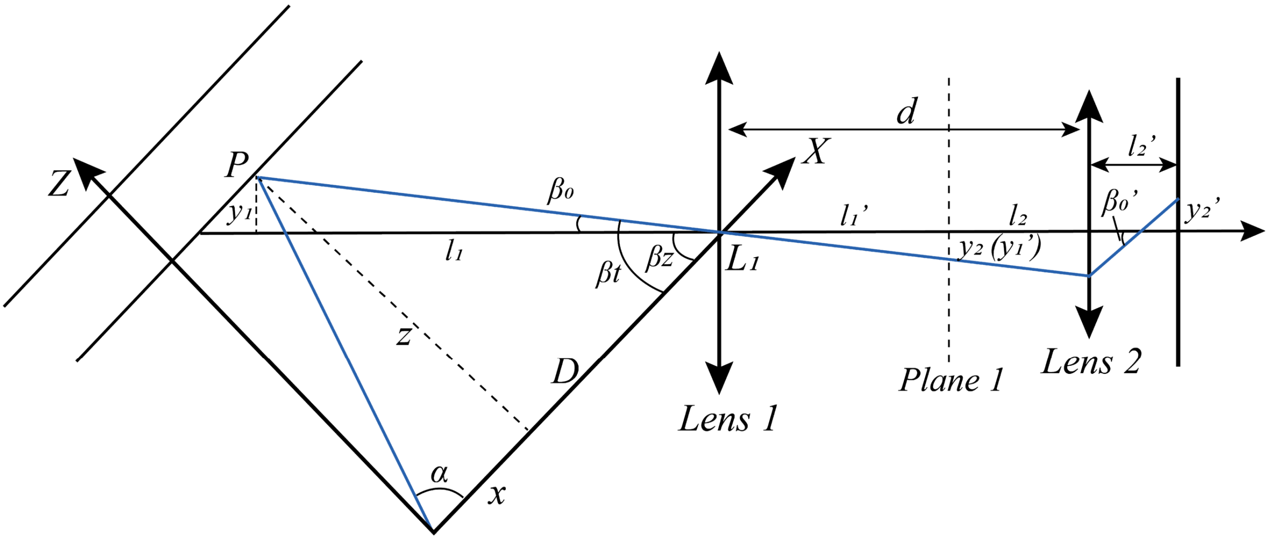


**Supplementary Fig. 13 │ 2D geometric schematic of the designed imaging system.** The light beam $PL_1$ reflected by the object is collected and refracted onto the angular detection plane through lens 1 and lens 2. The plane 1 is the primary image plane.

The goal of parameter optimization is to minimize the change of $y_2'$ with distance $z$ and the maximum change of $\beta_0'$ with $z$. Therefore, we analyzed the relationship between $\delta y_2'/\delta z$ and $\delta\beta_0'/\delta z$ and the system parameters $D$, $\alpha$, $\beta_z$, $d$, $f_1$ and $f_2$ (**Supplementary Fig. 14**). Considering the resolution and the detectable range of distance, we set the system parameters as $D$ = 50 mm, $\alpha$ = 90°, $\beta_z$ = 78°, $f_1$ = 75 mm, $f_2$ = 25mm, and $d$ = 145 mm. Optimal imaging parameters are listed in Table S1.

**Table S1. Optimal parameters for imaging 500 mm distance range**

| Imaging parameters | $\beta_0$ (degree) | $\boldsymbol{\beta_0'}$ **(degree)** | $y_1'$ (mm) | $\boldsymbol{y_2'}$ **(mm)** |
|---|---|---|---|---|
| z = 500 mm | 6.2894 | **28.5180** | -9.7266 | **6.9476** |
| z = 500.1 mm | 6.2905 | **28.5218** | -9.728 | **6.9486** |
| z = 700 mm | 7.9144 | **33.5093** | -11.6873 | **8.3481** |

When the distance $z$ is changed by 0.1 mm, the angle $\beta_0'$ of the light incident on the detector changes by approximately 0.0038°, and the light spot moves on the detector by approximately 1 μm, which is indistinguishable for a conventional CCD with a pixel size of 3–10 μm. By attaching the light-field imaging film onto the CCD, the distance change of 0.1 mm can be differentiated by angle detection. Under optimized

system parameters, a distance change of 200 mm causes the spot on the detector to move by 1.4 mm. It should be noted that the selection of imaging parameters depends largely on the distance $z$, so the system parameters must be determined according to the distance range of the application.

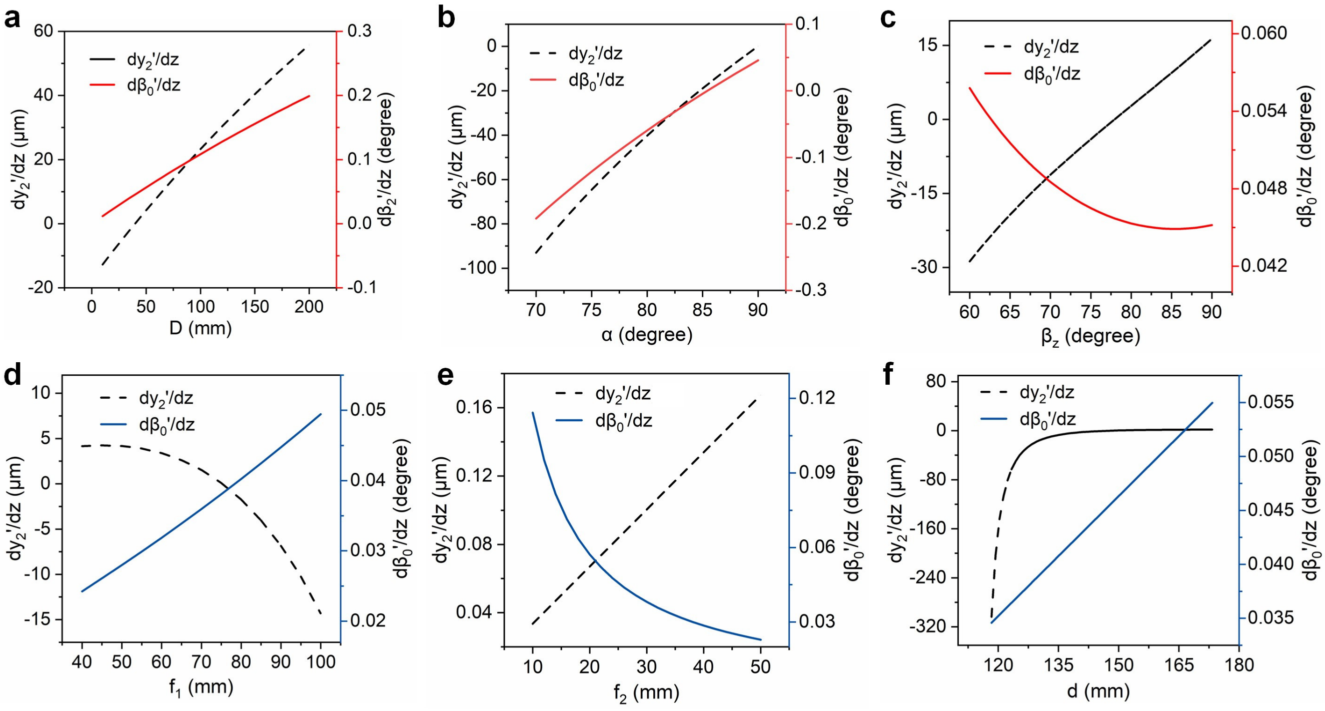


**Supplementary Fig. 14 │ Relationship between δ$y_2$'/δ$z$ and δ$\beta_0$'/δ$z$ and system parameters $D$, $\alpha$, $\beta_z$, $d$ and $f$.** δ$y_2$'/δ$z$ and δ$\beta_0$'/δ$z$ versus $D$ (**a**), $\alpha$ (**b**), $\beta_z$ (**c**), $f_1$ (**d**), $f_2$ (**e**), and $d$ (**f**).

**S7. Quantitative analysis of the 3D imaging system.**

*Quantitative relationship between depth of field and depth precision.*

According to the principle of triangulation ranging, the farther the object is from the imaging system, the smaller the angle change caused by the depth change and the lower the depth precision. The detectable depth range depends on the dynamic range of the angle measurement and the imaging system parameters. Under the designed system parameters ($D$ = 50 mm, $\alpha$ = 90°, $\beta_z$ = 78°, $f_1$ = 75 mm, $f_2$ = 25 mm, and $d$ = 145 mm), the incident angle on the azimuth detector varies from -30.2° to 40.4° when the detection distance varies from 200 mm to 2500 mm. When the angular resolution of the azimuth detector is 0.02°, the distance accuracy varies from 0.01 mm to 19.7 mm (**Supplementary Fig. 15**).

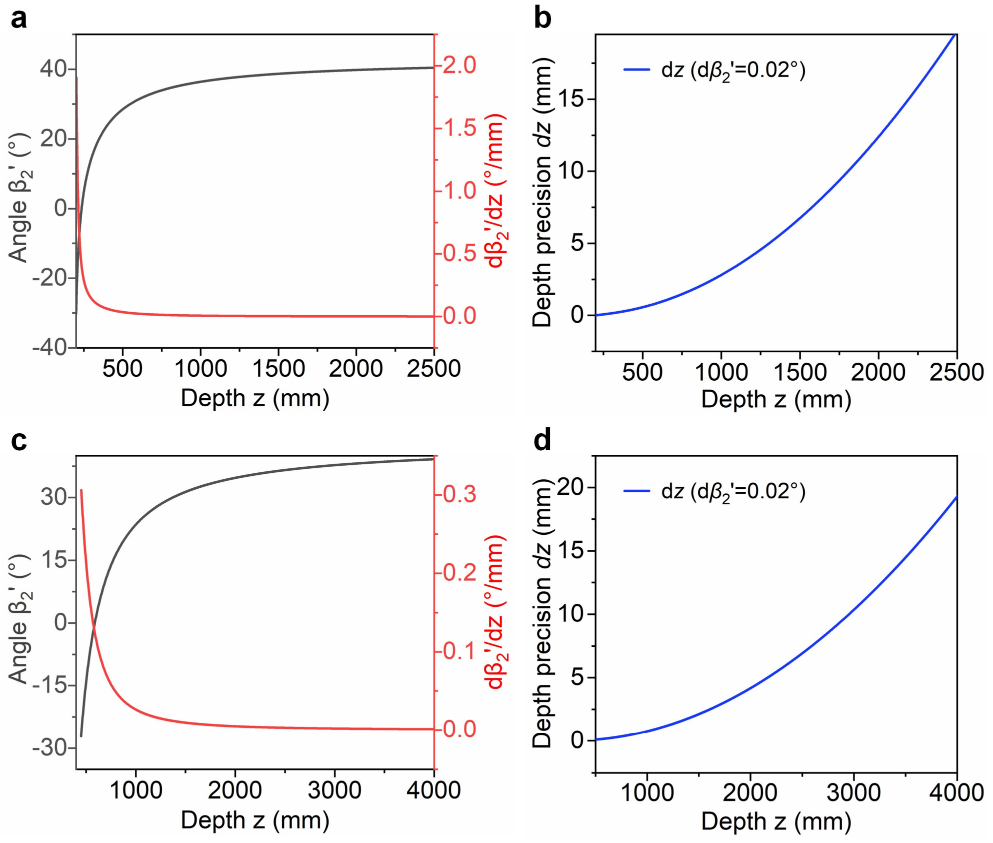


**Supplementary Fig. 15 │ Quantitative relationship between depth of field and depth precision. a**, Theoretical relationship between the imaging depth and the angle incident on the azimuth detector for the given system parameters ($D$ = 50 mm, $\alpha$ = 90°, $\beta_z$ = 78°, $f_1$ = 75 mm, $f_2$ = 25 mm, and $d$ = 145 mm). **b**, Depth precision versus imaging depth $z$. **c**, Theoretical relationship between the imaging depth and the angle incident on the azimuth detector for the given system parameters ($D$ = 125 mm, $\alpha$ = 90°, $\beta_z$ = 78°, $f_1$ = 100 mm, $f_2$ = 30 mm, and $d$ = 170 mm). **d**, Depth precision versus imaging depth $z$.

With system parameters ($D$ = 125 mm, $\alpha$ = 90°, $\beta_z$ = 78°, $f_1$ = 100 mm, $f_2$ = 30 mm, and $d$ = 170 mm), the incident angle on the azimuth detector varies from -27.2° to 39.2° when the detection distance varies from 450 mm to 4000 mm. When the angular resolution of the azimuth detector is 0.02°, the distance accuracy varies from 0.07 mm to 19.3 mm. For a depth of 1000 mm, the depth precision under both systems is 2.8 mm and 0.76 mm, respectively. To achieve a suitable depth of field and depth precision, different system parameters must be selected according to equations 18–26.

*Quantitative relationship between angular resolution and depth precision.*

There is a quantitative relationship between angular resolution and depth precision, depending on system parameters and imaging depth $z$ (**Supplementary Fig. 16**).

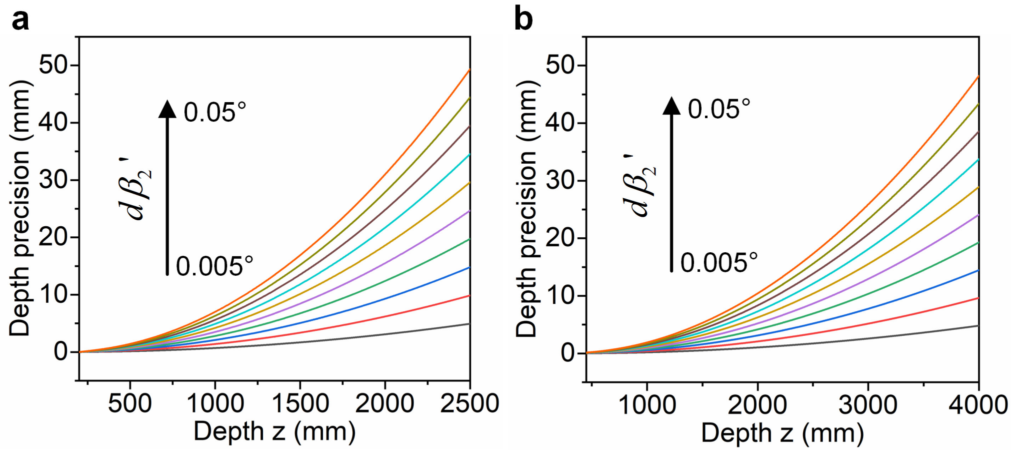


**Supplementary Fig. 16 │ Quantitative relationship between angular resolution and depth precision. a**, System parameters $D$ = 50 mm, $\alpha$ = 90°, $\beta_z$ = 78°, $f_1$ = 75 mm, $f_2$ = 25 mm, and $d$ = 145 mm. **b**, System parameters $D$ = 125 mm, $\alpha$ = 90°, $\beta_z$ = 78°, $f_1$ = 100 mm, $f_2$ = 30 mm, and $d$ = 170 mm. d$\beta_2$' denotes the angular resolution.

*Experimental results of the relationship between depth accuracy and depth of field*

Under the designed system parameters ($D$ = 50 mm, $\alpha$ = 90°, $\beta_z$ = 78°, $f_1$ = 75 mm, $f_2$ = 25 mm, and $d$ = 145 mm), the depth of field ranges from 200 mm to 2500 mm. We tested the depth accuracy of a plate sample at distances of 500 mm, 1000 mm, and 2000 mm, respectively (**Supplementary Fig. 17a)**. Under the designed system parameters ($D$ = 125 mm, $\alpha$ = 90°, $\beta_z$ = 78°, $f_1$ = 100 mm, $f_2$ = 30 mm, and $d$ = 170 mm), the depth of

field ranges from 450 mm to 4000 mm. We tested the depth accuracy of a plate sample at distances of 1000 mm, 2000 mm, and 3000 mm respectively (**Supplementary Fig. 17b)**.

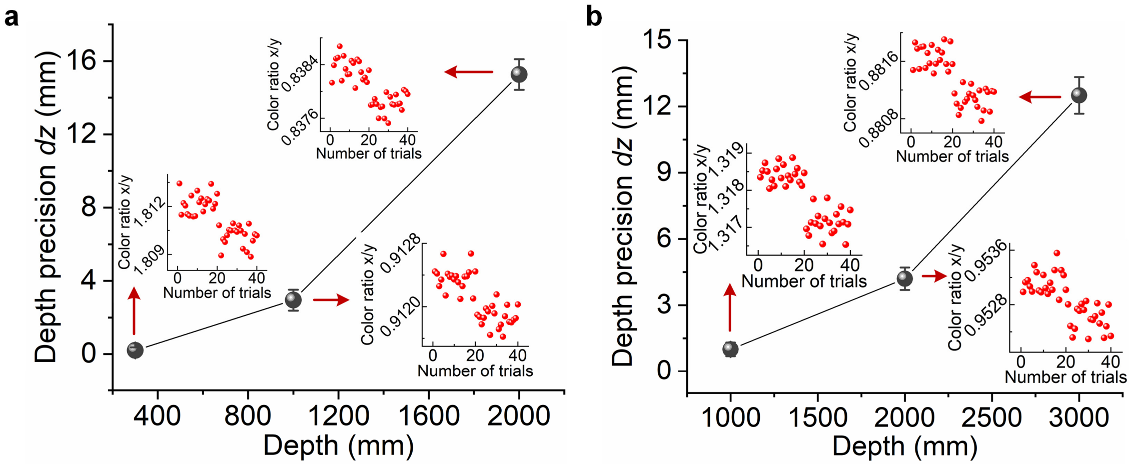


**Supplementary Fig. 17 |** Experimental results of the relationship between depth accuracy and depth of field. **a**, Under the designed system parameters ($D$ = 50 mm, $\alpha$ = 90°, $\beta_z$ = 78°, $f_1$ = 75 mm, $f_2$ = 25 mm, and $d$ = 145 mm), the depth accuracy of a plate sample at distances of 500 mm, 1000 mm, and 2000 mm, respectively. The insets show the color ratios corresponding to the minimum resolved depths at each depth. The three insets are for (300 mm, 300.2 mm), (1000 mm, 1002.8 mm), and (2000 mm, 2015.2 mm), respectively. Twenty measurement trials were performed for each depth, and data are presented as mean values +/- SEM. **b**, Under the designed system parameters ($D$ = 125 mm, $\alpha$ = 90°, $\beta_z$ = 78°, $f_1$ = 100 mm, $f_2$ = 30 mm, and $d$ = 170 mm), the depth accuracy of a plate sample at distances of 1000 mm, 2000 mm, and 3000, mm respectively. The three insets are for (1000 mm, 1001 mm), (2000 mm, 2004.2 mm), and (3000 mm, 3012.5 mm), respectively. Twenty measurement trials were performed for each depth, and data are presented as mean values +/- SEM.

## S8. Calibration of the 3D imaging system

*Calibration of the emission angle of multiline structured light*

The system uses an optical grating after a light source to generate multiline structured light and scans the object surface in a normal incidence mode. The angle between the two edge light planes of the structured light is $\alpha$, the angle between each structured light plane and the XOY plane is $\alpha_i$, and the angle between the structured light planes is $w$ (**Supplementary Fig. 18**). Since the structured light is incident perpendicular to the target, $\alpha_i$ can be obtained by the following formula:

$$\alpha_i = 90° \pm nw \tag{27}$$

Where $n$ represents the number of the line-structured light plane.

In the actual calibration, the structured light was vertically incident on a white flat plate, $OO'$ is the optical axis of the light source, and points $A$, $B$, $C$, and $D$ were the four corner points of the edge light strip on the surface. Points $A'$, $B'$, $C'$, and $D'$ are the four corner points of the edge light strip after the flat plate moves a certain distance. The coordinates of $A$, $B$, $C$, $D$, $A'$, $B'$, $C'$, and $D'$ are measured, and the plane equations of plane AA'D'D and plane BCC'B' can be established in the Cartesian coordinate system:

$$\begin{aligned} a_1x + b_1y + c_1z + d_1 = 0 \\ a_2x + b_2y + c_2z + d_2 = 0 \end{aligned} \tag{28}$$

Then the angle $\alpha$ between the two edge light planes of the structured light is:

$$\cos\alpha = \frac{|a_1a_2 + b_1b_2 + c_1c_2|}{\sqrt{a_1^2 + b_1^2 + c_1^2}\sqrt{a_2^2 + b_2^2 + c_2^2}} \tag{29}$$

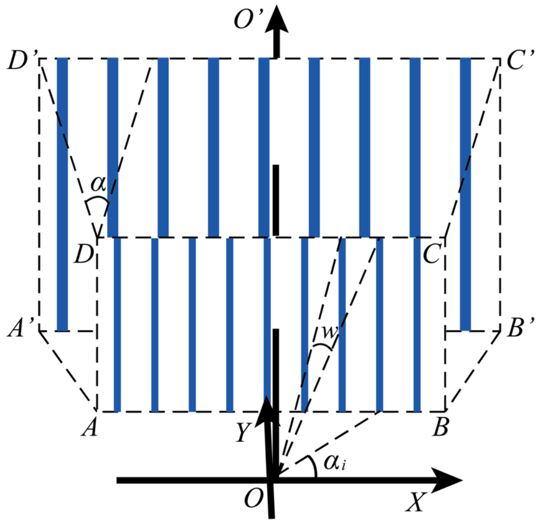


**Supplementary Fig. 18 | Schematic of the calibration of a multiline structured light source.** $\alpha$ is the angle between the AA'D'D plane and the BB'C'C plane, $w$ is the angle between the structured light planes, and $\alpha_i$ is the angle between each structured light plane and the XOY plane.

*Calibration of homemade camera parameters*

The conversion formula of the world coordinate system ($x_w$, $y_w$, $z_w$) and the pixel coordinate system ($u$, $v$) of the CCD is:

$$s\begin{bmatrix} u \\ v \\ 1 \end{bmatrix} = \mathbf{K}_{3\times3}\begin{bmatrix} \mathbf{R} & \mathbf{T} \\ 0 & 1 \end{bmatrix}\begin{bmatrix} x_w \\ y_w \\ z_w \\ 1 \end{bmatrix} \tag{30}$$

Where $s$ is the scale factor, **K** is the internal parameter matrix of the camera, **R** is the rotation matrix of the camera in the world coordinate system, and **T** is the translation matrix. The Zhang calibration method was used to determine internal parameters, external parameters, and distortion parameters of the camera. First, we printed a piece of paper with a black and white grid, and then took several images of the paper from different angles with the camera to be calibrated. Further, the camera calibration library toolbox_calib of Matlab was used to identify and process the feature points in the collected images to obtain the internal and external parameters as well as the distortion parameters of the camera.

## S9. Calibration of the 3D light-field sensor

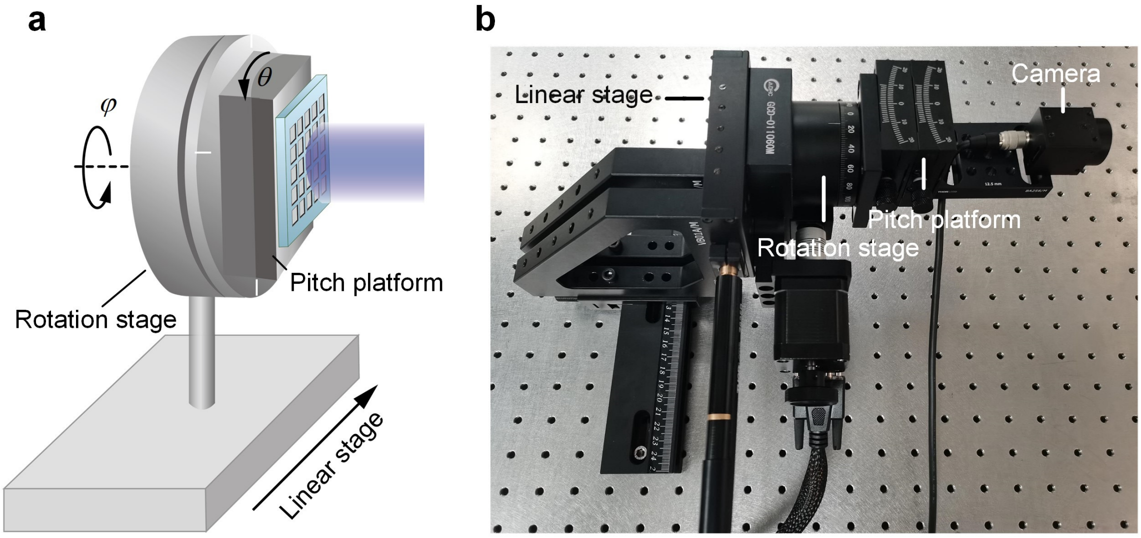


**Supplementary Fig. 19 | Calibration setup of the 3D light-field sensor.** Schematic (**a**) and optical photograph (**b**) of calibration setup for 3D light-field sensor arrays using a collimated LED source and multiple stages to rotate the image sensor in the $\theta$ and $\phi$ direction.

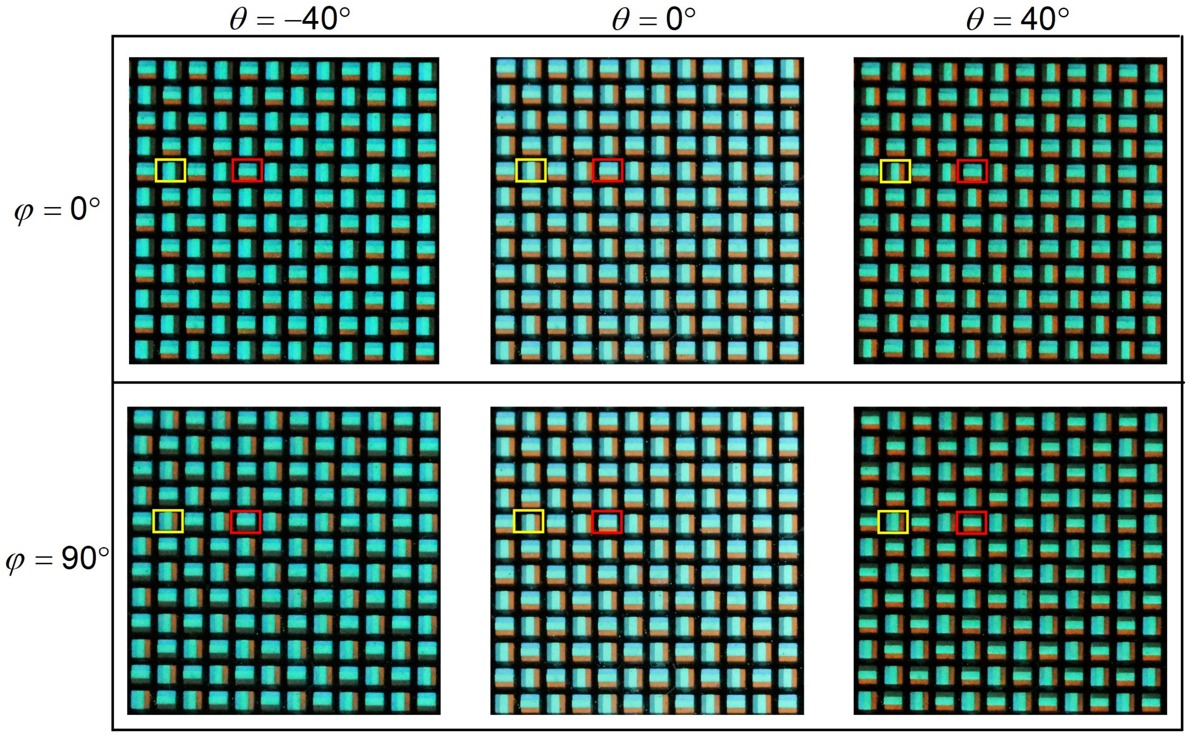


**Supplementary Fig. 20 | Example of raw color images taken at different incident angles**. When $\theta$ increases from -40° to 40° with $\varphi$ = 0°, the blueness of the pixel in the yellow square gradually fades. When $\theta$ increases from -40° to 40° with $\varphi$ = 90°, the blueness of the pixel in the red square becomes gradually weaker.

**S10. Factors affecting angular resolution**

We further conducted an analysis of the relationship between angular resolution and optical power as well as the X-ray dose rate (**Supplementary Fig. 21**). The results show that angular resolution is independent of the excitation power as long as it is not particularly low, since the intensity does not affect color tristimulus values. A weak excitation, however, results in a decrease in angular resolution. By measuring the direction of light at 405 nm and 0.5 mW, an angle resolution of approximately 0.01° can be obtained. It should be noted that the optical power is the set power of the laser and not the absolute power that actually hits the detector. When the thickness of the material layer is greater than 100 μm, perovskite nanocrystals can generate visible radioluminescence that is strong enough to be detected by the CCD or color sensors when irradiated at a dose rate of 10 μGy/s. When measuring the direction of X-rays at a dose rate of 10 μGy/s, the angular resolution is approximately 0.013°. The sensitivity of the angle measurement over the entire dynamic range was analysed. At a low light power (< 0.3 mW), the detection sensitivity of 60-degree incident light is attenuated by approximately 75% compared with the detection of normal incident light.

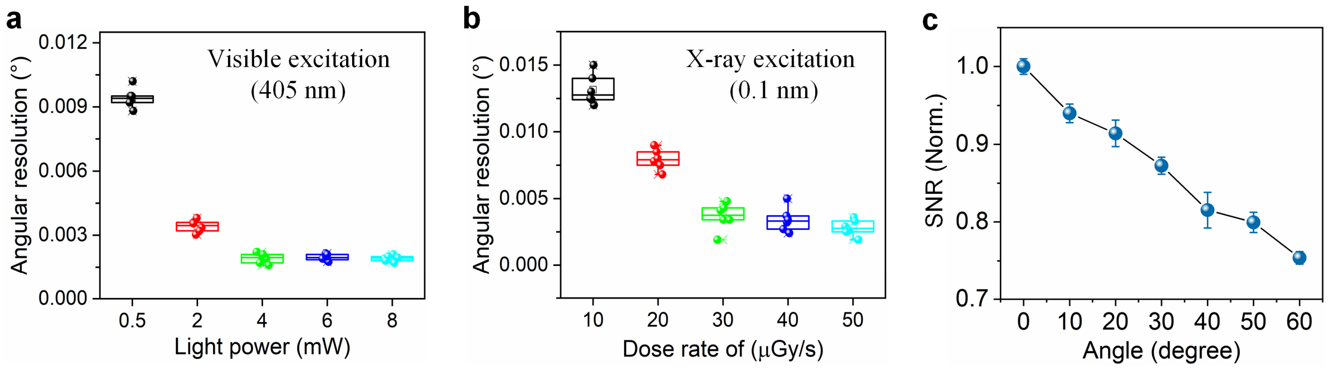


**Supplementary Fig. 21 | Analysis of factors influencing angular resolution.** Angular resolution of visible light (**a**) and X-rays (**b**) versus excitation power. Six measurements were performed at each light power and dose rate. The minima, maxima, centre, bounds of the box and whiskers, and percentiles are marked in boxplots. **c**, Sensitivity versus angle of incident light. The curve was measured at an incident optical power of 0.3 mW. Twenty measurements were performed at each angle, and data are presented as mean values +/- SEM.

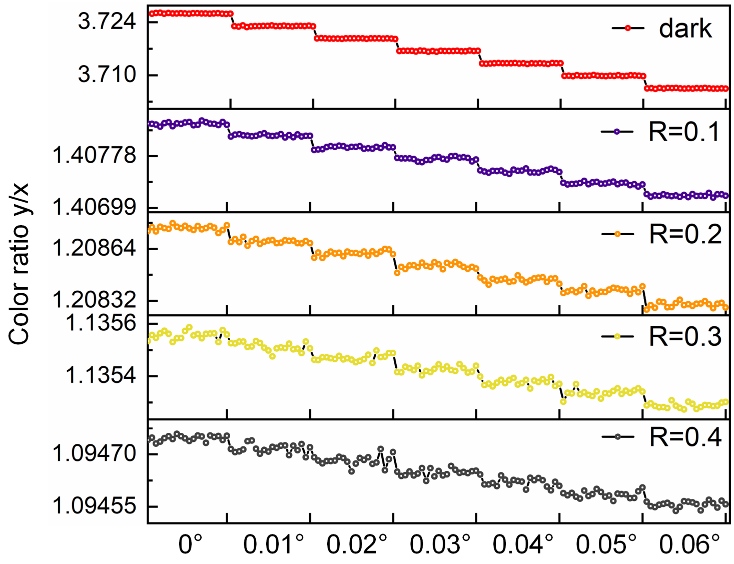


**Supplementary Fig. 22 │Effect of background light on angular resolution.** R represents the power ratio between the background light and the light source used in the 3D imaging system.

The intensity of the background light affects the depth precision when the 3D imaging system is used in natural light or incandescent light (**Supplementary Fig. 22**). If the ratio between the intensity of the background light and the light source used in the system exceeds 40%, the angular resolution of the azimuth detector will be worse than 0.01°. Therefore, to maintain high depth precision in daylight, a bandpass filter is placed in front of the azimuth detector array, which transmits only the wavelength of the light source.

## S11. Imaging procedure

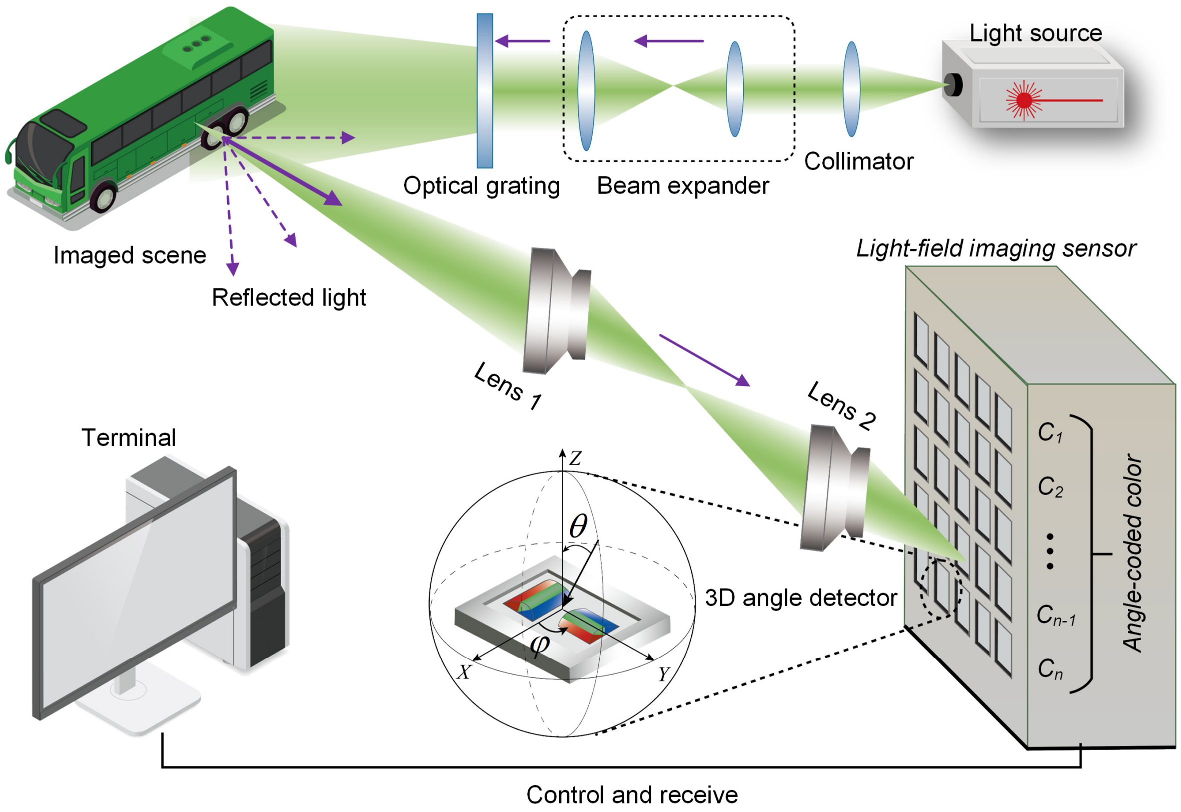


**Supplementary Fig. 23 │ Experimental setup.** A multiline structured light source (405 nm, Shenzhen Infrared Laser Technology Co., Ltd.) was generated by collimating and extending a light through a combination of a collimator and beam expander to an optical grating. An objective lens, consisting of lens 1 with a focal length of 100 mm and lens 2 with a focal length of 25 mm, collects the light reflected by the object and transmits it to the 3D light-field sensor comprising perovskite nanocrystal arrays.

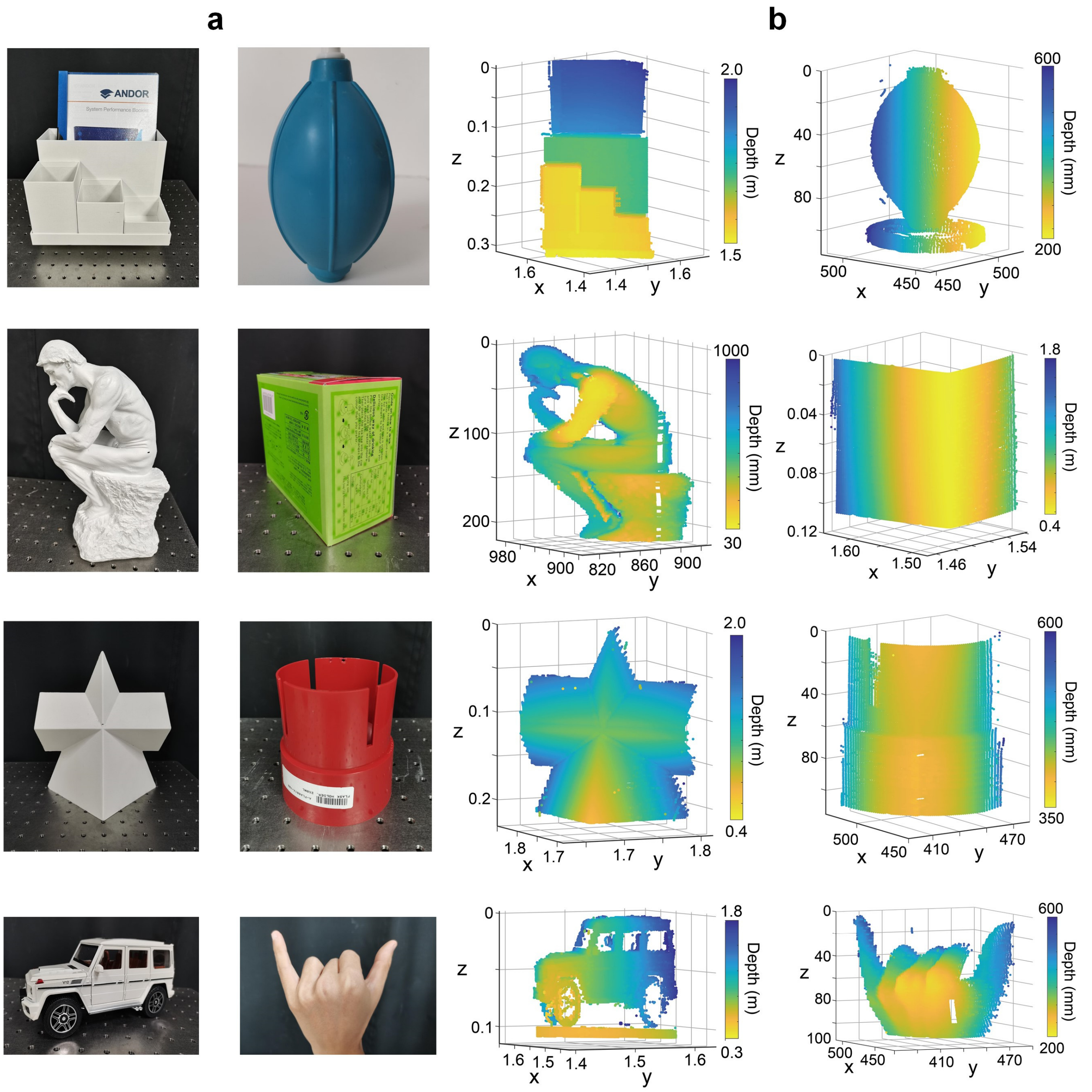


**Supplementary Fig. 24 │ 3D Imaging of scenes by pixelated color conversion**. **a,** Optical photographs of imaged scenes. **b,** 3D images of scenes captured using the 3D light-field sensor based on perovskite nanocrystal arrays. The colormap indicates the distance from the imaging point to the z-axis at the origin (x = 0, y = 0).

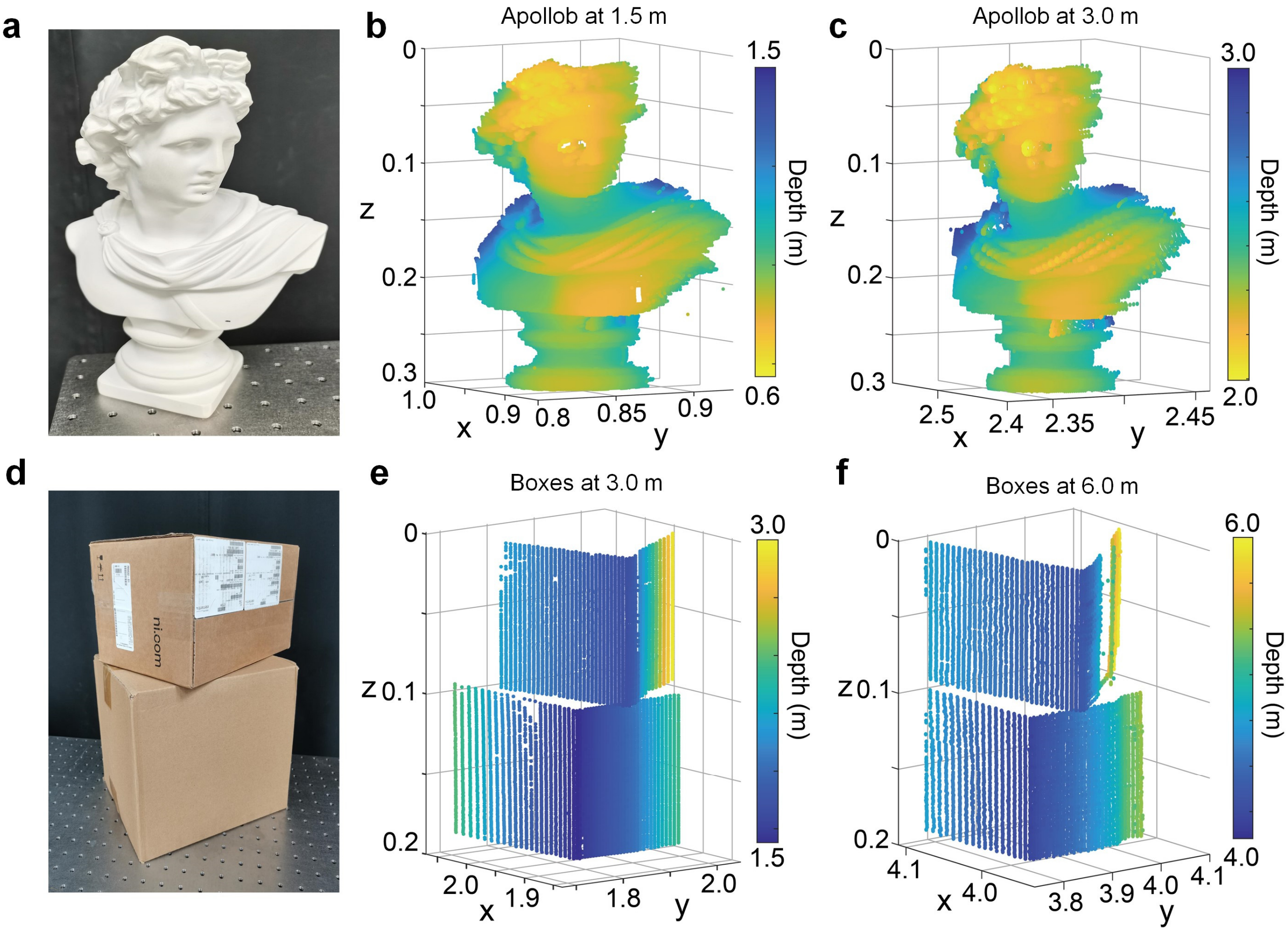


**Supplementary Fig. 25 │ 3D imaging of scenes at increasing depth by pixelated color conversion**. **a-c**, Optical photograph and the corresponding 3D images of a sculpture at 1.5 m and 3 m distances. **d-f**, Optical photograph and the corresponding 3D images of two boxes at 3.0 m and 6.0 m distances.

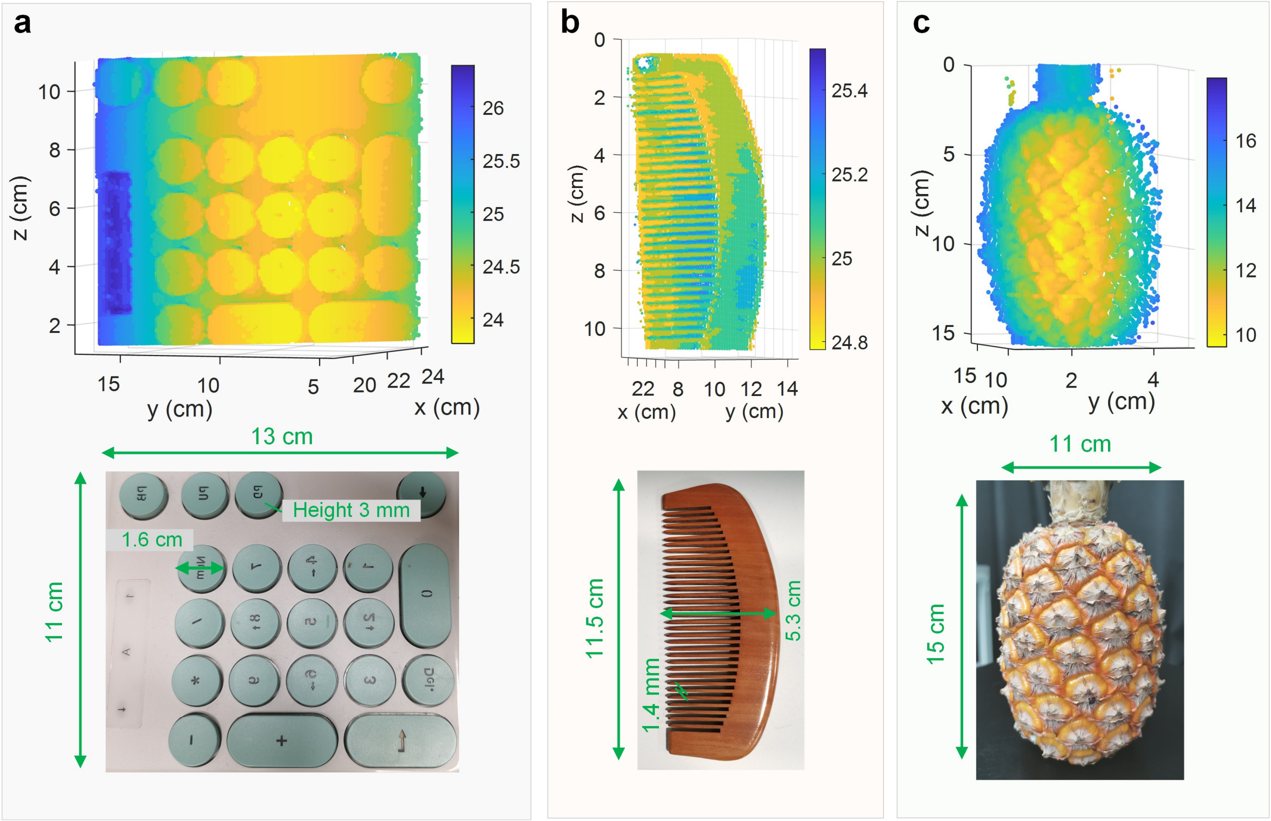


**Supplementary Fig. 26 │ 3D imaging of scenes with fine structure by pixelated color conversion**. **a-c**, Optical photographs and 3D depth images of scenes captured with the 3D light-field sensor based on perovskite nanocrystal arrays. The colormap indicates the distance from the imaging point to the z-axis at the origin (x = 0, y = 0).

## S12. Wavefront detection principle

Wavefront detection for extreme ultraviolet (EUV) light or X-rays typically uses Hartmann (e.g., Shack-Hartmann) wavefront sensing techniques in which a beam passes through a hole array (e.g., microlens array) and is projected onto a CCD camera that detects the beam sampled from each hole (e.g., microlens). The positions of individual point centroids are then measured and compared with reference positions. This enables the wavefront's local slopes to be measured at a large number of points by the following formula:

$$\tan\theta_{ij}^{x} = \frac{\Delta x_{ij}}{L}, \tan\theta_{ij}^{y} = \frac{\Delta y_{ij}}{L} \tag{31}$$

In our light-field sensor-based wavefront measurements, the local slope of the wavefront is directly obtained by the angle detectors without the need for an array of apertures or microlenses.

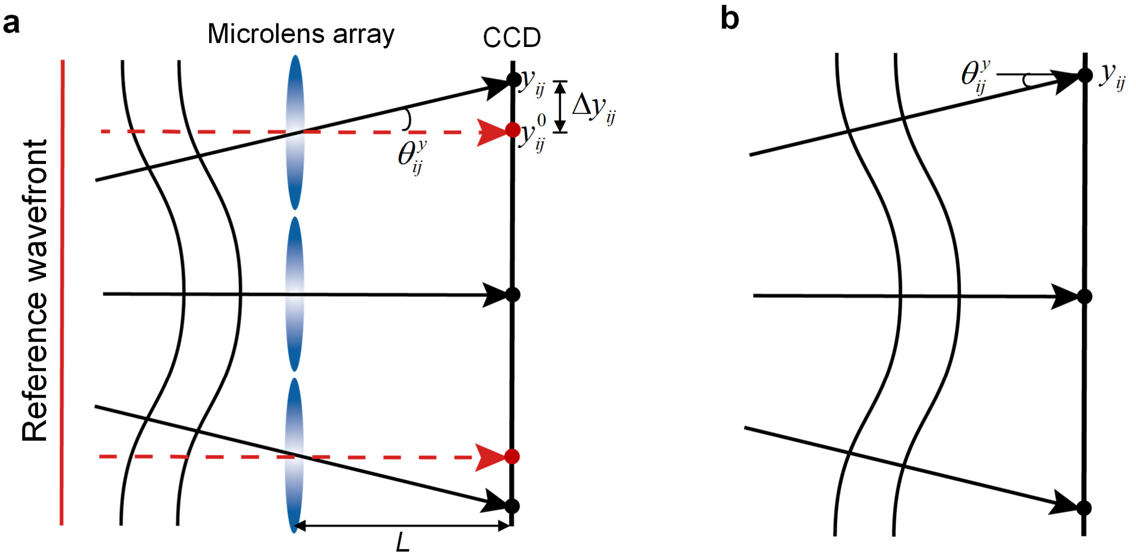


**Supplementary Fig. 27 | Schematic diagram of wavefront measurement. a,** Principle of Hartmann or Shack-Hartmann wavefront sensing. The distance deviation $\Delta y_{ij}$ between the spot centroids of the detected light and the reference light on the CCD is used to determine the wavefront's local slopes at position $y_{ij}$. **b,** Principle of wavefront measurement with the 3D light-field sensor. The wavefront's local slopes at position $y_{ij}$ are directly determined from the angle $\theta_{ij}^{y}$ detected by the corresponding angle detector of the sensor.

The wavefront's local slopes can be written according to the following expressions:

$$\begin{cases} \tan\theta_{ij}^{x} = \dfrac{dW(x_{ij}^{0}, y_{ij}^{0})}{dx} = \dfrac{\lambda}{2\pi}\dfrac{d\varphi(x_{ij}^{0}, y_{ij}^{0})}{dx} \\ \tan\theta_{ij}^{y} = \dfrac{dW(x_{ij}^{0}, y_{ij}^{0})}{dy} = \dfrac{\lambda}{2\pi}\dfrac{d\varphi(x_{ij}^{0}, y_{ij}^{0})}{dy} \end{cases} \tag{32}$$

where $W(x, y)$ represents the optical path difference and $\varphi\,(x, y)$ is the spatial phase. Integration of the measured derivative function enables reconstruction of the incident beam wavefront.

## S13. Spherical X-ray wavefront measurement.

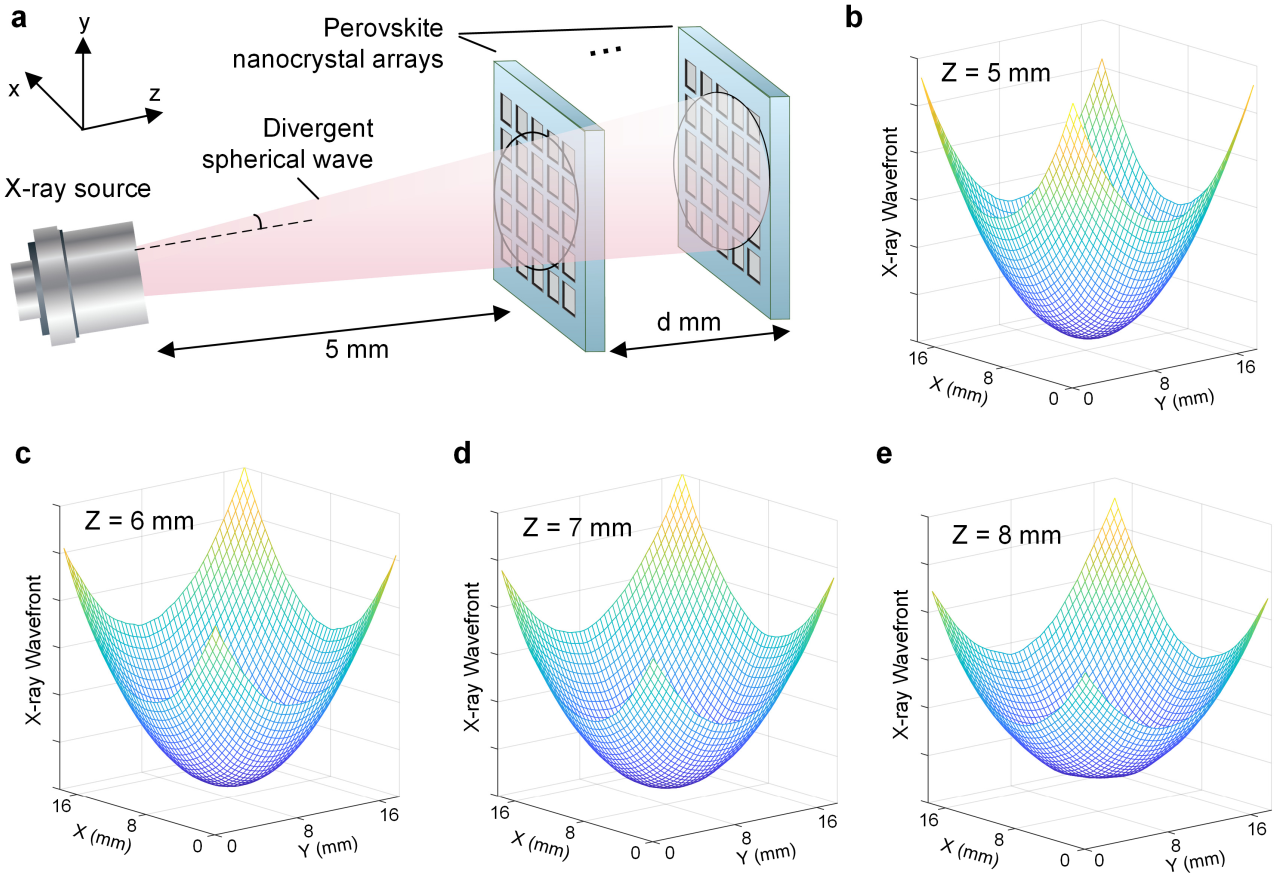


**Supplementary Fig. 28 │ Experimental setup and results of spherical wavefront measurement with perovskite nanocrystal arrays**. **a,** Experimental setup for wavefront measurement based on perovskite nanocrystal imaging arrays. **b-e**, Measured wavefront at z = 5 mm, 6 mm, 7 mm, and 8 mm, respectively.

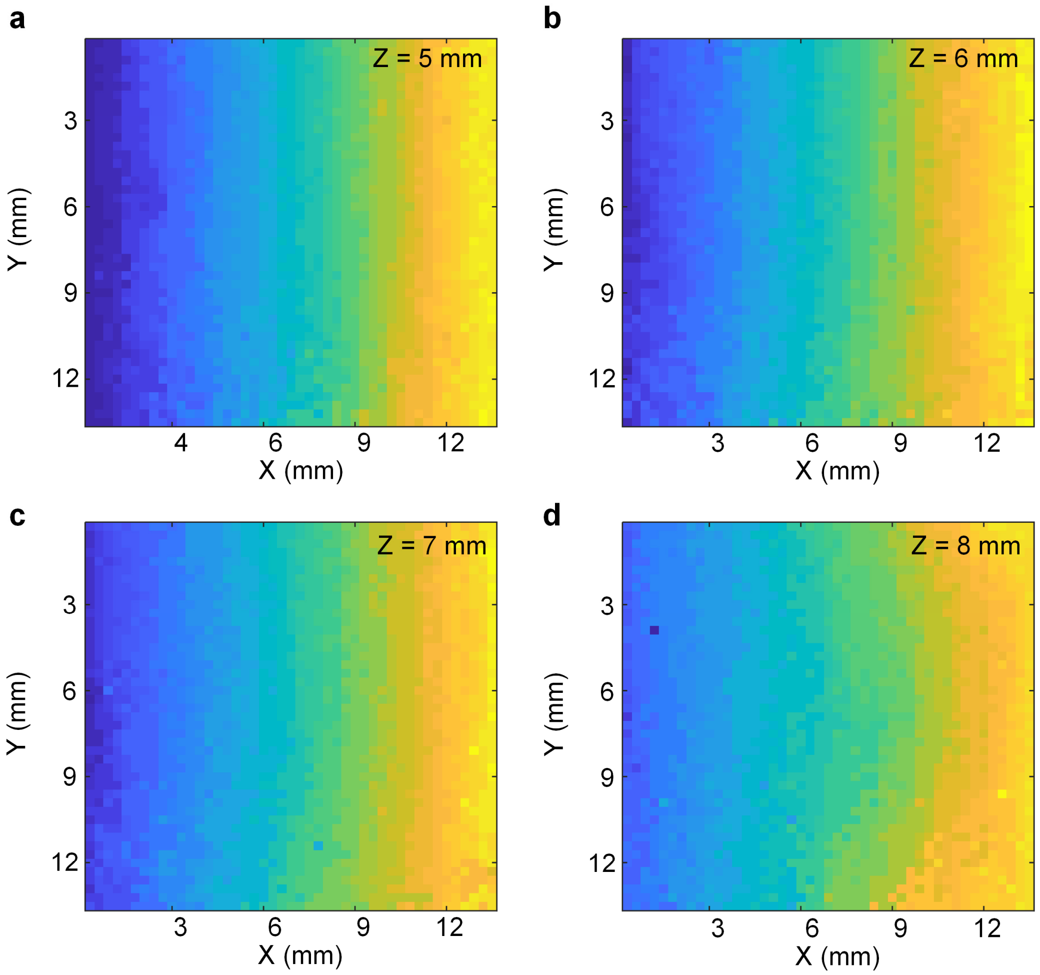


**Supplementary Fig. 29 │ a-d, Slope mapping in the X direction at z = 5 mm, 6 mm, 7 mm, and 8 mm, respectively.**

It should be noted that, as a proof of principle, we simply used color data mapping at z = 5 mm as calibration data to reconstruct the wavefronts at other distances z, so the measurement resolution of the slope becomes worse as the distance z increases. For practical applications, sufficient calibration angle sampling points must be obtained to ensure the angular resolution of the wavefront reconstruction.

## S14. References

43. Berger, M.J. XCOM: Photon Cross Sections Database (NIST, 2013); https://www.nist.gov/pml/xcom-photon-cross-sections-database.
